# Exploiting sweet relief for preeclampsia by targeting autophagy-lysosomal machinery and proteinopathy

**DOI:** 10.1038/s12276-024-01234-x

**Published:** 2024-05-17

**Authors:** Zheping Huang, Shibin Cheng, Sukanta Jash, Jamie Fierce, Anthony Agudelo, Takanobu Higashiyama, Nazeeh Hanna, Akitoshi Nakashima, Shigeru Saito, James Padbury, Jessica Schuster, Surendra Sharma

**Affiliations:** 1 Department of Pediatrics, Women and Infants Hospital of Rhode Island, Warren Alpert Medical School of Brown University, Providence, RI 02905 USA; 2 HAYASHIBARA Co.,Ltd., 675-1 Fujisaki, Naka-ku, Okayama, Japan; 3 Division of Neonatology, Department of Pediatrics, New York University Long Island School of Medicine, Mineola, New York, NY USA; 4 Department of Obstetrics and Gynecology, Faculty of Medicine, University of Toyama, Toyama, Japan; 5 Department of Pediatrics, University of California, San Francisco, CA USA; 6 Present Address: Department of Obstetrics and Gynecology, University of Texas Medical Branch, Galveston, TX 77555 USA

**Keywords:** Medical research, Drug discovery

## Abstract

The etiology of preeclampsia (PE), a severe complication of pregnancy with several clinical manifestations and a high incidence of maternal and fetal morbidity and mortality, remains unclear. This issue is a major hurdle for effective treatment strategies. We recently demonstrated that PE exhibits an Alzheimer-like etiology of impaired autophagy and proteinopathy in the placenta. Targeting of these pathological pathways may be a novel therapeutic strategy for PE. Stimulation of autophagy with the natural disaccharide trehalose and its lacto analog lactotrehalose in hypoxia-exposed primary human trophoblasts restored autophagy, inhibited the accumulation of toxic protein aggregates, and restored the ultrastructural features of autophagosomes and autolysosomes. Importantly, trehalose and lactotrehalose inhibited the onset of PE-like features in a humanized mouse model by normalizing autophagy and inhibiting protein aggregation in the placenta. These disaccharides restored the autophagy-lysosomal biogenesis machinery by increasing nuclear translocation of the master transcriptional regulator TFEB. RNA-seq analysis of the placentas of mice with PE indicated the normalization of the PE-associated transcriptome profile in response to trehalose and lactotrehalose. In summary, our results provide a novel molecular rationale for impaired autophagy and proteinopathy in patients with PE and identify treatment with trehalose and its lacto analog as promising therapeutic options for this severe pregnancy complication.

## Novel therapeutic approach for preeclampsia: targeting autophagy pathways

Autophagy, a process in cells that helps maintain organ growth and balance, is important for the immune system and stress response. However, when autophagy doesn’t work properly, it can lead to diseases, including preeclampsia, a serious pregnancy issue. Dr. Surendra Sharma and his team at the Women and Infants Hospital of Rhode Island discovered that trehalose, a natural sugar, can fix autophagy and restore the ability of the cell’s waste disposal system. They tested this on human cells and a mouse model of preeclampsia. The results showed that trehalose and a similar sugar, lactotrehalose, effectively fixed autophagy, stopped harmful protein buildup, and reduced the production of harmful factors associated with preeclampsia. This suggests that sugars like trehalose could be potential treatments for preeclampsia. “This summary was initially drafted using artificial intelligence, then revised and fact-checked by the author.”

## Introduction

Autophagy is a major molecular process that maintains cellular and organ developmental homeostasis^1–3^. Autophagy also plays a key role in the development of the immune system and the physiological response to stress^4,5^. Impaired autophagy is associated with various human diseases, including neurodegenerative diseases, certain types of cancers, immune disorders, and the process of aging^6–8^. We recently demonstrated that impaired autophagy and autophagy-induced proteinopathy play a core etiological role in preeclampsia (PE), a severe complication of pregnancy^9–16^.

PE is a multifactorial, human pregnancy-specific disorder characterized by the new onset of hypertension and either proteinuria or end-organ dysfunction in 5–8% of all pregnancies at or after 20 weeks of gestation^17–19^. This condition is associated with a high incidence of maternal and fetal morbidity and mortality and is considered a major risk factor for chronic diseases later in life, including cardiovascular disease, diabetes, renal disease, and possibly dementia^20–22^. Early detection of PE and effective treatment remain challenging. The only effective treatment is delivery of the placenta. The etiology of PE remains unknown, and its clinical manifestations are unpredictable^19^. Poor placentation, defective spiral artery remodeling, endoplasmic reticulum (ER) and oxidative stress, nitric oxide dysregulation, hypoxia, ischemia, local and systemic inflammation, and an imbalance of proangiogenic and antiangiogenic factors are all thought to contribute to the pathophysiology of PE^22–25^. Research on these etiological factors has not yet identified any definite treatment modalities. Based on our observations of impaired autophagy and protein aggregation^9–16^, we speculated that these pathologies could be targeted for promising treatment options. To advance this line of investigation, we recently developed a novel blood test to detect protein aggregates for PE and Alzheimer’s disease (AD) using an autophagy-deficient human trophoblast cellular model^16^. This assay sensitively detects aggregates composed of several proteins, including transthyretin (TTR), amyloid β1-42 (Aβ42), and *cis* P-tau, in the serum of patients with PE.

Recent efforts have been devoted to identifying new drugs or repurposing drugs for an array of human diseases that are associated with autophagic deficiency. Trehalose is a naturally occurring, nonreducing disaccharide that can act as a chaperone and an autophagic activator and can correct autophagy and restore the degradative capacity of the lysosomal biogenesis machinery by activating the transcription factor of the E box (TFEB)^26–28^. Notably, trehalose is currently considered a drug of choice for treating neurodegenerative diseases characterized by impaired autophagy and misfolded proteins, including AD, Parkinson’s disease, Huntington’s disease, and amyotrophic lateral sclerosis (ALS)^29–32^. Given the ability of trehalose to restore autophagy-lysosomal biogenesis, we sought to evaluate the biological effects of trehalose in inhibiting PE-associated pathological characteristics using both in vitro cellular models and an in vivo humanized mouse model of PE. One caveat with the clinical use of trehalose may be its susceptibility to degradation by the gut enzyme trehalase^33^. Thus, we also evaluated its lacto analog, lactotrehalose, which has been shown to be resistant to degradation by trehalase^33^.

## Methods

### Human study

All protocols concerning the use of human material were approved by the Institutional Review Board of Women and Infants Hospital (WIH03-0096). All the subjects provided informed consent prior to participating in the study. The current study included the use of serum and placental samples from pregnant women. Placental samples were obtained from pregnant women with early-onset PE (e-PE) (<34 gestational weeks) and gestational age-matched pregnant women. The ACOG definition was used to define early-onset and late-onset PE. The exclusion criteria included chronic hypertension, gestational or preexisting diabetes, fetal demise, daily tobacco use, fetal anomalies, and multiple gestations. For placental sample collection, a 1 cm^3^ specimen was removed from the placenta and vigorously washed with chilled phosphate-buffered saline solution. After any additional blood was removed from the placental tissue, a portion was stored at −80 °C until further use. All methods were carried out in accordance with relevant guidelines and regulations. Demographic information is provided in Supplementary Table 1.

### Animal study

All animal protocols were approved by the Lifespan Institutional Animal Care and Use Committee (IACUC: 505722). We established a humanized mouse model of PE using serum from PE patients and injected i.p. into pregnant IL-10^−/−^ mice^34^. IL-10 is a pregnancy-compatible cytokine, and IL-10 deficiency increases the susceptibility of pregnant mice to severe pregnancy complications. IL-10^−/−^ mice on a C57BL/6 background were obtained from the Jackson Laboratory (Bar Harbor, ME) and housed in a pathogen-free facility at Rhode Island Hospital. All the mice used in this study were 7–8 weeks old. The experimental procedures are shown in Fig. 5a. The day of vaginal plug appearance was considered gestational day (gd) 0. Severe preeclampsia serum (PES) or normal pregnant serum (NPS) were collected as previously reported^34^. A total of 100 µl of serum (NPS or PES) was intraperitoneally (i.p.) injected into each mouse on the morning of gd 10 to induce PE or serve as a control. Trehalose or other sugars were i.p. administered to the mice via one injection on gd 9 (prevention), 2 injections on gd 12 and gd 14 (treatment), or 3 injections on gd 9, gd 12 and gd 14 at a dose of 2 g/kg of mouse body weight. Lactotrehalose was administered on gd 9, gd 12, and gd 14 at the same dose. On gd 16, the mice were transferred to individual metabolic cages. In pilot experiments, we tested a dose range between 0.5 g/kg and 2 g/kg trehalose and lactotrehalose for intraperitoneal administration in pregnant mice. A dose of 2 g/kg induced the strongest protective effects without any apparent maternal or fetal toxicity. We carried out most intraperitoneal administration experiments with a dose of 2 g/kg. On gd 17, urine was collected and stored at −80 °C. creatinine ELISA (ELISA kit for measurement of mouse urinary albumin from Exocell, Philadelphia, PA) was used to measure the albumin levels. A creatinine EIA kit (ELISA kit from Quidel Corporation, San Diego) was used for the measurement of urinal creatinine levels. Proteinuria is represented as the ratio of urinary albumin to creatinine. Systolic blood pressure was recorded with an established tail-cuff method using a DigiMed blood pressure analyzer (MicroMed, Louisville, KY). Mice were euthanized on gd 17, and kidney, serum, and placenta were collected for further analysis. For evaluation of fetal growth restriction, fetal weight was measured.

### Reagents used

Antibodies were purchased from commercial sources, and the details are provided in Supplementary Table 2. A ProteoStat Aggresome Detection Kit (ENZO, ENZ-51023-KP002, 1:2000) was used to detect aggregated proteins as previously described^16^. Trehalose was purchased from Sigma (Sigma, T9531), and lactotrehalose was a kind gift from Hayashibara Co., Ltd. (Okayama, Japan). Human and Mouse VEGFR1/Flt-1 Quantikine ELISA Kits were obtained from R&D Systems (#DVR100C, MVR100). FITC-trehalose was purchased from Ximbio (London, England).

### RT‒PCR

Quantitative real-time polymerase chain reaction (qRT‒PCR) was performed with total RNA isolated from primary trophoblast cells exposed to normoxia, hypoxia, or hypoxia + trehalose (50 mM) for 48 h. The experiment was carried out according to the manufacturer’s instructions, and TRIzol and RNeasy Mini Kit columns (Qiagen, 74104) were used. RNA integrity (RIN) was evaluated using an Agilent 2100 Bioanalyzer (Agilent Technologies, California, USA). All the samples had an average RIN ranging from 9.3 to 9.7. RNA was quantified using a Nanodrop ND-2000 spectrophotometer (Thermo Fisher Scientific, CA, USA). Following the manufacturer’s instructions, 1 µg of RNA was used to generate complementary DNA (cDNA) using a High-Capacity cDNA Reverse Transcription Kit (Applied Biosystems^TM^). PowerTrack™ SYBR Green Master Mix (Applied Biosystems, A46012) was utilized to conduct quantitative PCR in accordance with the guidelines provided by the manufacturer. This study utilized the following human primers: CTSD Forward 5′ TGCTCAAGAACTACATGGACGC 3′, CTSD Reverse 5′ CGAAGACGACTGTGAAGCACT 3′. LAMP1 Forward 5′ CAGATGTGTTAGTGGCACCC 3′, LAMP1 Reverse 5′ TTGGAAAGGTACGCCTGGATG 3′, Ribosomal protein 13a (RPL13a) Forward 5′ GCCCTACGACAAGAAAAAGCG 3′, RPL13a Reverse 5′ TACTTCCAGCCAACCTCGTGA 3′.

### Autophagy-proficient and autophagy-deficient first trimester trophoblast cell lines

Autophagy-proficient and autophagy-deficient human first-trimester extravillous trophoblast cell lines were established by stable transfection with PMRX-IRES-puro-mStrawberry and pMRX-IRES-puro-mStrawberry-Atg4B^C74A^, an Atg4B^C74A^ mutant expression vector that inhibits MAP1LC3B-II formation, respectively, as previously described^10^. The control vector pMRX-IRES-puro-mStrawberry only encodes monomeric red fluorescent protein.

### Cell culture and hypoxia-reoxygenation (H/R) treatment

Human primary trophoblast cells were purchased from ScienCell Research Laboratories (Carlsbad, CA) and cultured in TM supplemented with 5% FBS, trophoblast growth supplement, and 1% penicillin/streptomycin solution at 37 °C in a 5% CO_2_ atmosphere (all reagents were purchased from ScienCell Research Laboratories, Carlsbad, CA). The third trimester extravillous trophoblast cell line (TCL1) and autophagy-deficient and autophagy-proficient trophoblast cell lines were cultured in RPMI media supplemented with 10% FBS, 100 U/ml penicillin, and 100 µg/ml streptomycin (Gibco, 15140) at 37 °C in a 5% CO_2_ atmosphere. The cells were synchronized in FBS, washed with Dulbecco’s phosphate-buffered saline (DPBS) before treatment, and incubated with reagents in serum-free media. Cells from passages 2 to 3 were used. Briefly, cells in serum-free media (in the presence of different concentrations of trehalose, lactotrehalose, other saccharides, or vehicle) were exposed for 3 days to normoxia in a regular cell incubator or 1% O_2_ in a hypoxia chamber (1% O_2_, 5% CO_2_, and 94% N_2_) (Thermo Electron, Marietta, OH, USA). On Day 3, before harvesting the cells, hypoxia-treated cells were incubated for 3 h under normoxic conditions for reoxygenation treatment. During normoxia or hypoxia exposure, cells were treated with trehalose or lactotrehalose in a dose-dependent manner (0–50 mM) or at a fixed dose (50 mM). A similar approach was used for treatment with baflomycin A1 (Baf A1), and autophagy was restored by trehalose.

### Immunostaining

The immunostaining procedures for placental tissue have been described in detail elsewhere^11,13^. Sections were stained according to the manufacturer’s instructions, and images were captured with a Zeiss microscope.

For the cell culture experiments, primary trophoblasts or TCL1 cells were grown on coverslips, exposed to normoxia or H/R for 3 days in serum-free medium, and then fixed in 4% formalin for 20 min. The fixed cells were washed in PBS and incubated in blocking buffer, followed by the procedures described above (excluding the incubation with Sudan blue). After immunofluorescence staining, the sections or cells on the coverslips were mounted with anti-quenching mounting medium containing DAPI (Vector Laboratories, Inc., Burlingame, CA) and observed using a Nikon Eclipse 80i fluorescence microscope (Tokyo, Japan) with a Texas Red filter set for Alexa 594-conjugated antibodies and a FITC filter set for Alexa 488-conjugated antibodies. Images from both control and treated cells were acquired using identical settings. Images were processed with brightness/contrast adjustment using Photoshop (CS6, https://www.adobe.com/) using the same settings.

### Detection of aggregated proteins in the mouse placenta and trophoblast cells

A ProteoStat Aggresome Detection Kit (ENZO) was used to detect aggregated proteins in cells or sections according to a modified protocol described previously^16^. Briefly, placental sections were first deparaffinized and then treated with 0.1% Sudan blue for 20 min at room temperature. After that, the sections were fixed with 4% formaldehyde in PBS for 15 min at 37 °C, followed by intensive washing in deionized water. Finally, the sections were stained with ProteoStat dye at a 1:2000 dilution for 3 min at room temperature. Destaining by incubating sections in 1% acetic acid was performed to remove nonspecific background. Nuclei were stained with DAPI. For detection of protein aggregates in cultured cells, primary human trophoblasts or TCL1 cells, the cells were plated on glass coverslips, exposed to normoxia or H/R, fixed at 72 h, and then stained with ProteoStat dye for 15 min as described above. Autophagy-deficient or autophagy-proficient trophoblast cells were exposed to normoxia or H/R for 48 h and then fixed and stained as described above.

### Dual staining using immunofluorescence and ProteoStat rotor dye

Normoxia- or H/R-treated primary human trophoblasts on coverslips were immunostained with a primary antibody against human TTR or beta-amyloid and a secondary antibody, Alexa Fluor 488 donkey anti-rabbit IgG, followed by staining with ProteoStat dye as described above. The cells were washed three times, mounted and observed under a microscope (Nikon, A1R, Japan) using a Texas Red filter set for the ProteoStat dye and a FITC filter set for Alexa Fluor 488-conjugated antibodies. All images were acquired with a 20× objective lens. Negative controls were generated by replacing the primary antibody with purified rabbit IgG or mouse IgG. The figures were processed with brightness/contrast adjustment using Photoshop (CS6, https://www.adobe.com/) with the same settings.

### Transmission electron microscopy

Ultrathin 70-nm-thick sections were cut and double-stained with uranyl acetate and lead citrate. For quantification of autophagic vacuoles (autophagosomes and autolysosomes), photomicrographs showing the perinuclear area from hypoxia-treated (*n* = 20) or control cells (*n* = 20) (21000 magnification) were randomly taken using a Philips 410 transmission electron microscope.

### Western blotting

Cells were lysed in RIPA buffer. Cell lysates were separated using 4–12% mini-PROTEAN® TGX Stain-Free™ Precast Gels according to standard procedures (Bio-Rad), and Western blotting was performed as previously described^11–14,25,34^. For separation under nonreducing conditions, protein extracts were mixed with sample buffer that did not contain reducing agents or SDS; the samples were not heated and then separated in running buffer containing 25 mM Tris, 192 mM glycine, and 0.05% SDS. The density of the blots was measured using ImageJ (NIH).

### Calcineurin phosphatase activity assay

The phosphatase activity of calcineurin was measured using a Calcineurin Phosphatase Activity Assay Kit (colorimetric) (Abcam, ab139461) according to the manufacturer’s instructions.

### Transcriptomic profiling with RNA-seq

Mouse placentas were collected at gd17, processed by extensive washing with saline, and sent to BGI (Shenzhen, China) for total RNA isolation (TRIzol) and RNA sequencing. Total RNA was assessed for quality and quantified using a Nanodrop and Agilent Technologies 2100 bioanalyzer (Agilent Technologies, Santa Clara, CA, USA). mRNA molecules were purified using oligo(dT)-attached magnetic beads. The purified mRNA molecules were fragmented into small pieces. cDNA was synthesized with random primers, followed by end repair, 3’ adenylation, adapter ligation, and PCR amplification. Double-strand PCR products were denatured and circularized by the splint oligo sequence, followed by amplification with phi29 and sequencing via the DNBSEQ high-throughput platform. Sequencing reads were filtered using the software SOAPnuke developed by BGI and aligned to the reference genome using Bowtie 2^35^, and expression was quantitated for each sample using RSEM^36^. Differentially expressed genes (DEGs) were identified using DESeq2^37^. Statistically significant genes were defined based on a nominal *p* < 0.05. For identification of rescue genes, genes for which no samples had an FPKM greater than or equal to one were filtered out. A gene was identified as a rescue gene if the *p* value was <0.05 for PES vs. NPS or PES vs. “Treatment” and if the log base 2-fold change for each gene was greater than 1.

### Statistical analysis

All values are expressed as the mean ± SEM from at least four independent experiments. We performed statistical analysis using one-way or two-way ANOVA or two-tailed Student’s *t* tests in GraphPad Prism (version 9.3). Statistical significance was set at *p* < 0.05.

## Results

### Evidence for trehalose-mediated inhibition of protein aggregation in primary human trophoblasts exposed to hypoxia/reoxygenation

**Fig. 1:**
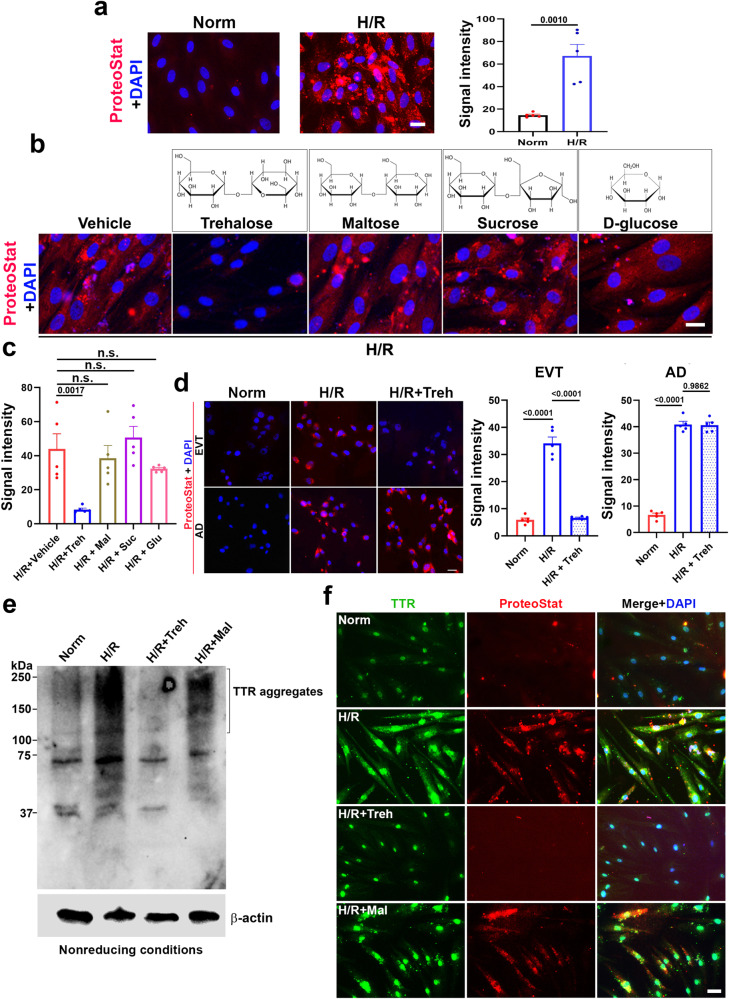
Trehalose inhibits hypoxia-reoxygenation (H/R)-induced protein aggregation in primary human trophoblasts (PHTs) in an autophagy-dependent manner. Cells (70% confluence) with or without trehalose (Treh) or other saccharides (50 mM) were exposed for 3 days to normoxia (Norm) or H/R and then fixed for staining or pelleting for Western blotting. The nuclei were stained with DAPI (blue). **a** ProteoStat staining and signal intensity analysis showed that PHTs exposed to H/R exhibited robust accumulation of protein aggregates compared to those in normoxia-treated cells. **b**, **c** Only trehalose prevented protein aggregation in response to H/R. **d** Trehalose significantly decreased H/R-induced protein aggregation in autophagy-proficient extravillous trophoblasts (EVT) but not in autophagy-deficient EVTs (AD). **e**, **f** Immunoblotting under nonreducing conditions (e) and dual staining (f) demonstrated that HPTs, when exposed to H/R, induced the accumulation of robust TTR compared to that under normoxia (as represented by the high-molecular-weight TTR bands in (**e**); trehalose, not maltose, inhibited TTR aggregates in H/R. Bars: 20 µm. The data are presented as the means ± SEMs, and statistical analysis was performed using Student’s *t* test (**a**) or one-way ANOVA adjusted for multiple comparisons (**c**, **d**). n.s. not significant. All images are representative of 5 independent experiments.

As described above, although trehalose has been described as an activator of autophagy^26–28^, no information is available on whether this nonmammalian disaccharide inhibits protein aggregation in trophoblasts in response to ER stressors such as hypoxia, a key pathological factor associated with PE. We first investigated whether trehalose could inhibit protein aggregation in hypoxia/reoxygenation (H/R)-exposed primary human trophoblasts using a novel method that we recently established^16^. H/R-induced protein aggregation was identified by the fluorescence signal of ProteoStat, a rotor dye, and was reversed by trehalose (Fig. 1a–c). To examine the dose-dependent effects of this treatment, we first demonstrated that trehalose reversed protein aggregation in a dose-dependent manner (see Supplementary Fig. 4c for trehalose). Next, we compared the ability of 50 mM trehalose with that of other disaccharides (maltose and sucrose) and a monosaccharide (D-glucose) to inhibit H/R-induced protein aggregation. Among these treatments, only trehalose inhibited protein aggregation in H/R-treated primary human trophoblasts (Fig. 1b, c). To assess the ability of trehalose to promote autophagy, we used autophagy-proficient and autophagy-deficient extravillous trophoblast cells (EVTs) that were established by stable transfection of the control vector and the ATG4B^C74A^ mutant gene, respectively (see Methods)^10^. Trehalose inhibited protein aggregation in the autophagy-proficient EVTs but not in the autophagy-deficient EVTs (Fig. 1d), suggesting that trehalose indeed requires the autophagic machinery for its effects.

To validate the findings described above, we utilized Western blotting under nonreducing conditions to directly separate protein aggregates in extracts from normoxic or H/R-exposed primary human trophoblasts in the absence or presence of 50 mM trehalose or maltose. We demonstrated that TTR is one of the proteins that undergoes protein aggregation in PE. As shown in Fig. 1e, a large amount of protein aggregates with high molecular weights were identified by the anti-TTR antibody in the H/R-exposed cells but not in the normoxia-treated cells. These high-molecular-weight TTR aggregates were not detected in the cells treated with trehalose. Maltose did not reduce the content of these aggregates (Fig. 1e). Similarly, the immunofluorescence staining pattern suggested that H/R induced TTR aggregation, as demonstrated by ProteoStat, and that this effect was significantly reduced by trehalose (Fig. 1f). Maltose again failed to inhibit protein aggregation. The ProteoStat and TTR signals colocalized to indicate the aggregation pattern of TTR (Fig. 1f). To complement these results, we also showed that Aβ42 aggregation was induced by H/R in primary human trophoblasts and inhibited by trehalose (Supplementary Fig. 1).

### Trehalose-mediated inhibition of protein aggregation is associated with the restoration of lysosomal biogenesis-related proteins in H/R-treated human trophoblasts

**Fig. 2:**
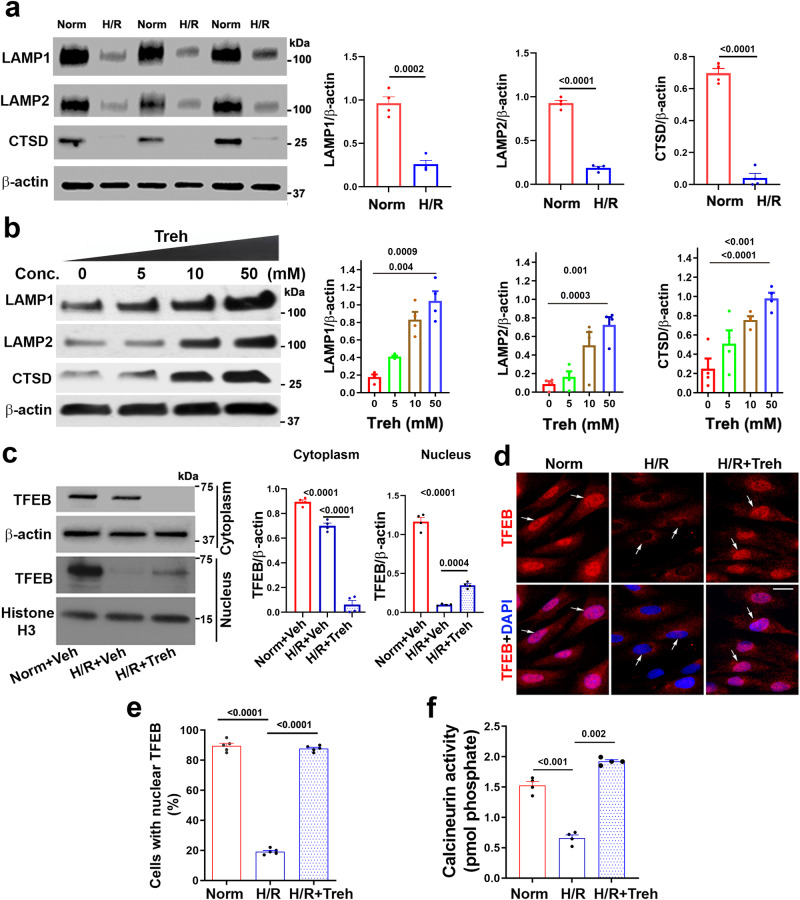
Trehalose reverses H/R-induced downregulation of LAMP1, LAMP2, and cathepsin D (CTSD), calcineurin activity, and inhibition of TFEB cytoplasmic translocation in primary human trophoblast cells. Cells were exposed to normoxia (Norm) or H/R for 3 days in the presence or absence of varying concentrations of trehalose. Then, the cells were lysed for Western blotting, calcineurin activity was measured, or the cells were fixed for immunofluorescence staining. **a** H/R exposure significantly reduced the expression of LAMP1, LAMP2, and CTSD. **b** Treh rescued the protein levels of LAMP1, LAMP2, and CTSD in the H/R-exposed trophoblasts in a dose-dependent manner (*n* = 4). **c** H/R reduced the abundance of TFEB in the nuclear fraction, which, however, was partly rescued by 50 mM Treh treatment. **d** Representative immunofluorescence images of H/R-exposed trophoblasts showing that exposure to H/R induces the cytoplasmic translocation of TFEB in PHTs, which was reversed by Treh treatment. Arrows indicate the nuclei. Bar: 20 µm. **e** The percentage of cells containing nuclear TFEB in (**d**) was calculated and plotted (*n* = 4) (*p* < 0.0001). **f** Treh reversed the H/R-induced decrease in calcineurin enzymatic activity (*p* < 0.0001). The data are presented as the mean ± SEM (*n* = 4) and were analyzed using Student’s *t* test (**a**) or one-way ANOVA adjusted for multiple comparisons (**b**, **c**, **e**, **f**). The calcineurin activity was normalized to the protein concentration in each treatment group.

We hypothesize that H/R exposure impairs autophagy and negatively affects the expression of lysosomal biogenesis-related proteins and that trehalose treatment restores the expression of these molecules. Western blotting analysis revealed that H/R significantly decreased the abundance of lysosomal-associated protein 1 (LAMP1), LAMP2, and cathepsin D (CTSD) compared to that in the control cells grown under normoxic conditions (Fig. 2a), and trehalose restored the expression of these proteins in a dose-dependent manner (Fig. 2b). Additionally, we evaluated whether H/R treatment of primary human trophoblasts dysregulated the nuclear translocation of the transcription factor of E box (TFEB), the master regulator of lysosomal biogenesis machinery. Cells were exposed to three conditions: normoxia + vehicle, H/R + vehicle, or H/R + trehalose (50 mM). The cells were processed to isolate nuclear or cytoplasmic fractions, followed by Western blotting for TFEB, β-actin and histone H3 or fixation for staining. The Western blotting results, shown in a representative experiment (*n* = 4), demonstrated that H/R treatment abrogated TFEB nuclear translocation and that trehalose significantly restored this process (Fig. 2c). TFEB immunodetection and nuclear DAPI staining revealed that cells grown under normoxia mostly showed TFEB nuclear localization, whereas H/R treatment led to TFEB translocation to the cytoplasm (Fig. 2d, e). Importantly, trehalose treatment of H/R-exposed cells restored the nuclear localization of TFEB.

Dephosphorylation of several transcription factors, such as nuclear factor of activated T cells (NFAT), for their nuclear translocation is mediated by the activation of the calcium-dependent phosphatase calcineurin^38,39^. To determine whether trehalose-mediated reversal of the effects of H/R (as shown in Fig. 2d, e) is the result of calcineurin activation, we compared calcineurin activity in response to the H/R and H/R + trehalose treatments in primary human trophoblast cells. The results (Fig. 2f) confirm the data presented in Fig. 2c–e and show that H/R inactivates calcineurin, whereas trehalose significantly restores calcineurin activity.

The inability of maltose to block the accumulation of protein aggregates may be correlated with its inability to restore lysosomal biogenesis in response to H/R. Consistent with this notion, the results in Supplementary Fig. 2a, b demonstrate that, unlike trehalose, maltose failed to reverse the H/R-mediated reduction in lysosomal proteins, including LAMP1 and CTSD. Maltose likewise had no effect on normoxia-treated cells (Supplementary Fig. 2a, b). In addition to primary human trophoblasts, we also used an immortalized third-trimester extravillous trophoblast cell line, TCL1, to evaluate trehalose-mediated activity against protein aggregation and the restoration of lysosomal biogenesis-related proteins. Our results showed that trehalose inhibited H/R-mediated accumulation of ProteoStat-positive protein aggregates (Supplementary Fig. 2c) and restored the levels of LAMP1, LAMP2, and CTSD in a dose-dependent manner in TCL1 cells (Supplementary Fig. 2d, e). Overall, these data demonstrate that the impaired autophagy and proteinopathy in H/R-exposed primary human trophoblasts/TCL1 cells is a result of dysregulation of lysosomal biogenesis, which can be reversed by trehalose. In addition, we validated our data by assessing whether trehalose can restore the levels of autophagic lysosomal biogenesis-related proteins inhibited by another autophagy inhibitor, bafilomycin A1 (Baf A1). In Supplementary Fig. 3, we show that trehalose (50 mM) counteracts the inhibitory effects of Baf A1 on LAMP1, LAMP2, and CTSD in exposed primary human trophoblasts, suggesting that this disaccharide can restore autophagy impaired by a variety of triggers.

### Trehalose normalizes the ultrastructural features of autophagy in primary human trophoblasts exposed to H/R

**Fig. 3:**
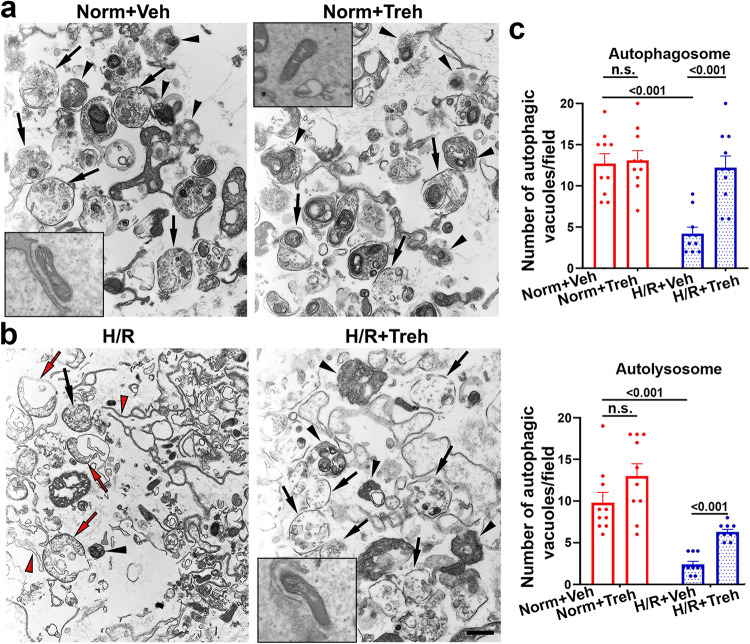
Treh treatment reversed the H/R-induced changes in ultrastructural features. Cells were exposed to normoxia (Norm) or H/R for 3 days in the presence of vehicle (Veh) or 50 mM trehalose (Treh) and then fixed for TEM. **a**, **b** Representative TEM images of cells subjected to normoxia (Norm **a**) or H/R (H/R **b**) with (right) or without Treh (left). Red arrows and arrowheads indicate deformed mitochondria and isolation membranes (left), respectively. The black arrows and arrowheads indicate intact autophagosomes and autolysosomes, respectively. **c** The numbers of autophagosomes and autolysosomes were counted and statistically compared. Bar: 600 nm (**a**, **b**). Images (**a**, **b**) are representative of 4 independent experiments. Inserts show images of normal mitochondria selected from different vision fields of the same sections. The data in (**c**), presented as the mean ± SEM, were statistically analyzed using two-way ANOVA coupled with a Bonferroni post hoc correction (*n* = 20, *p* < 0.001).

We used transmission electron microscopy (TEM)^40^ to validate the effects of disruption of H/R-mediated autophagy and its restoration by trehalose and to characterize the ultrastructural morphology of autophagic vacuoles in normoxic or H/R-treated trophoblasts as described previously^41^. There were significantly fewer autophagosomes and autolysosomes in the H/R-exposed primary human trophoblasts than in the normoxic trophoblasts (Fig. 3a–c). The H/R-treated cells exhibited a large number of phagophores, isolated membranes, and damaged mitochondria (Fig. 3b (left panel)). Trehalose treatment of H/R-exposed primary human trophoblasts reversed this effect and normalized the ultrastructural features of autophagosomes, autolysosomes, and mitochondria (Fig. 3b (right panel)), which were comparable to those of the normoxia+vehicle (Fig. 3a) and normoxia+trehalose (Fig. 3c) groups.

### Lactotrehalose, a trehalase-resistant trehalose analog, is an equally potent regulator of lysosomal biogenesis machinery, proteinopathy, and the production of the antiangiogenic factor sFlt-1

We examined the ability of lactotrehalose (see Supplementary Fig. 4a), a trehalase-resistant trehalose analog, to restore H/R-mediated inhibition of lysosomal biogenesis-related proteins such as LAMP1 and CTSD. Like trehalose, lactotrehalose increased the expression of these proteins in H/R-treated primary human trophoblasts in a dose-dependent manner (Supplementary Fig. 4b). In addition, LT significantly inhibited the accumulation of ProteoStat-positive protein aggregates (Supplementary Fig. 4c) in a dose-dependent manner. The ability of LT to restore LAMP1 and CTSD in TCL1 trophoblast cell lines is consistent with the observations in primary human trophoblasts (Supplementary Fig. 4d–f).

**Fig. 4:**
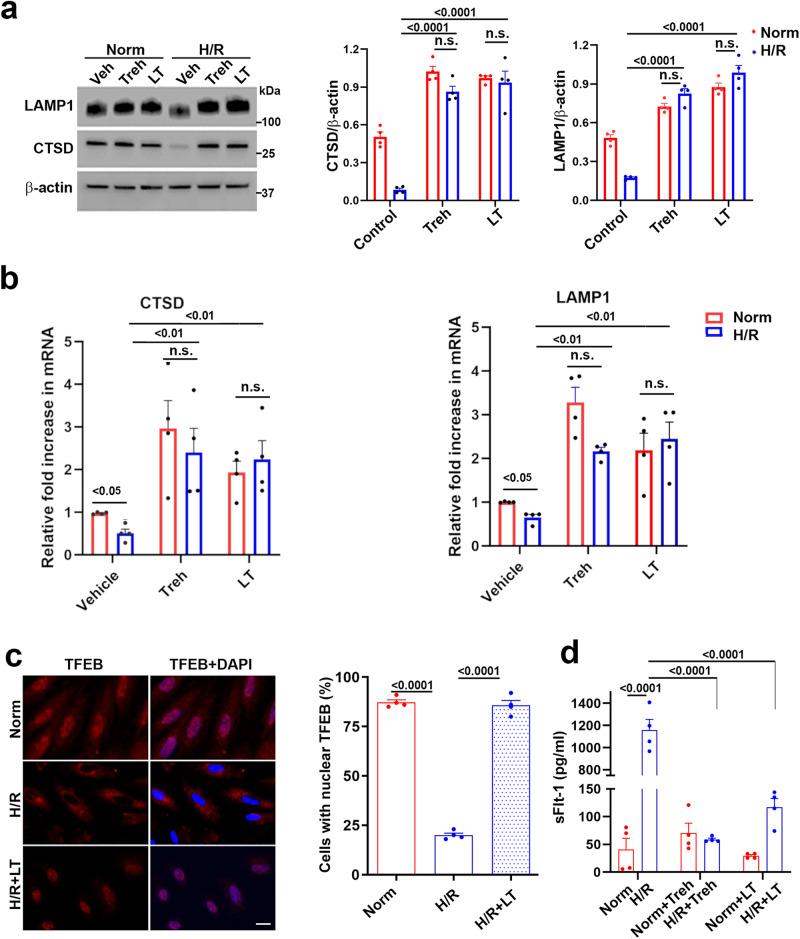
Lactotrehalose, like trehalose, reversed the H/R-induced downregulation of LAMP1, cathepsin D (CTSD), and nuclear TFEB and normalized sFlt-1 levels in PHTs. PHTs treated with vehicle, 50 mM Treh, or LT were exposed to normoxia (Norm) or hypoxia (H/R) for 3 days. Then, the cells were collected for Western blot analysis or fixed for staining, and the supernatants were collected for the sFlt1 assay. **a** Western blotting showed that, similar to Treh, LT (50 mM) rescued H/R-induced downregulation of LAMP1 and CTSD (*n* = 4). **b** RT‒PCR for LAMP1 and CTSD was performed as described in the Methods section. **c** Immunofluorescence staining (left) and quantification of cells with nuclear TFEB (right) showed that LT (50 mM) reversed the reduction in the number of cells with nuclear TFEB (red) induced by H/R. The nuclei (blue) were stained with DAPI. Bar: 20 µm. (*n* = 4). **d** sFlt-1 levels in the culture supernatants were detected using a commercial ELISA kit, normalized to the protein concentration, and then statistically compared among various treatments (*n* = 4). The data are presented as the means ± SEMs and were analyzed using two-way ANOVA (**a**, **b**, **d**) or one-way ANOVA (**c**) coupled with multiple comparisons.

Additionally, trehalose and lactotrehalose were equally potent at restoring LAMP1 and CTSD expression, which was significantly reduced by H/R treatment in human primary trophoblasts (Fig. 4a). To validate the Western blotting data, we also assessed the transcriptional regulation of LAMP1 and CTSD by hypoxia and the reversal of this process by trehalose and lactotrehalose (Fig. 4b). Our data suggested that hypoxia downregulated the expression of LAMP1 and CTSD, whereas trehalose and lactotrehalose significantly upregulated the expression of these genes and reversed the hypoxia-mediated downregulation (*n* = 4). Figure 4c shows that H/R treatment induced TFEB translocation to the cytoplasm, and lactotrehalose cotreatment reversed this translocation to the nucleus, as indicated by the colocalization of TFEB and DAPI in the nucleus. These data were quantified as the percentage of cells with nuclear TFEB and were statistically compared among the different treatment conditions. The quantitative results showed that the alterations in the subcellular distribution of TFEB under different treatment conditions were significant.

One of the consequential features of PE is increased placental and circulating expression of antiangiogenic factors such as sFlt-1^12,42^. The production of the antiangiogenic factor sFlt-1, which is increased in PE, was inhibited by both trehalose and lactotrehalose in H/R-treated primary human trophoblasts (Fig. 4d). These results show that trehalose and lactotrehalose not only restore autophagy and inhibit proteinopathy but also suppress the production of PE-associated antiangiogenic factors.

### Trehalose and lactotrehalose induce autophagy and inhibit proteinopathy in vivo and protect against PE

We previously established a human serum-based mouse model of PE that recapitulates all the features of the human disease^34^. We determined whether trehalose crosses the placental barrier by monitoring the movement of FITC-conjugated trehalose across the placenta in pregnant mice (*n* = 5) 6 h after intraperitoneal administration. FITC-conjugated trehalose was easily detected in the placenta by FITC immunofluorescence, particularly in the junctional zone (JZ) region (Supplementary Fig. 5). A magnified insert shows its localization mainly in the cytoplasmic area of cells costained with FITC and nuclear DAPI (Supplementary Fig. 5). The placental tissue is marked for specific regions, including the labyrinth (La), JZ, and decidua (De). We observed substantial staining in the JZ region. We have previously shown that protein aggregates accumulate mainly in the JZ region^12,13^.

**Fig. 5:**
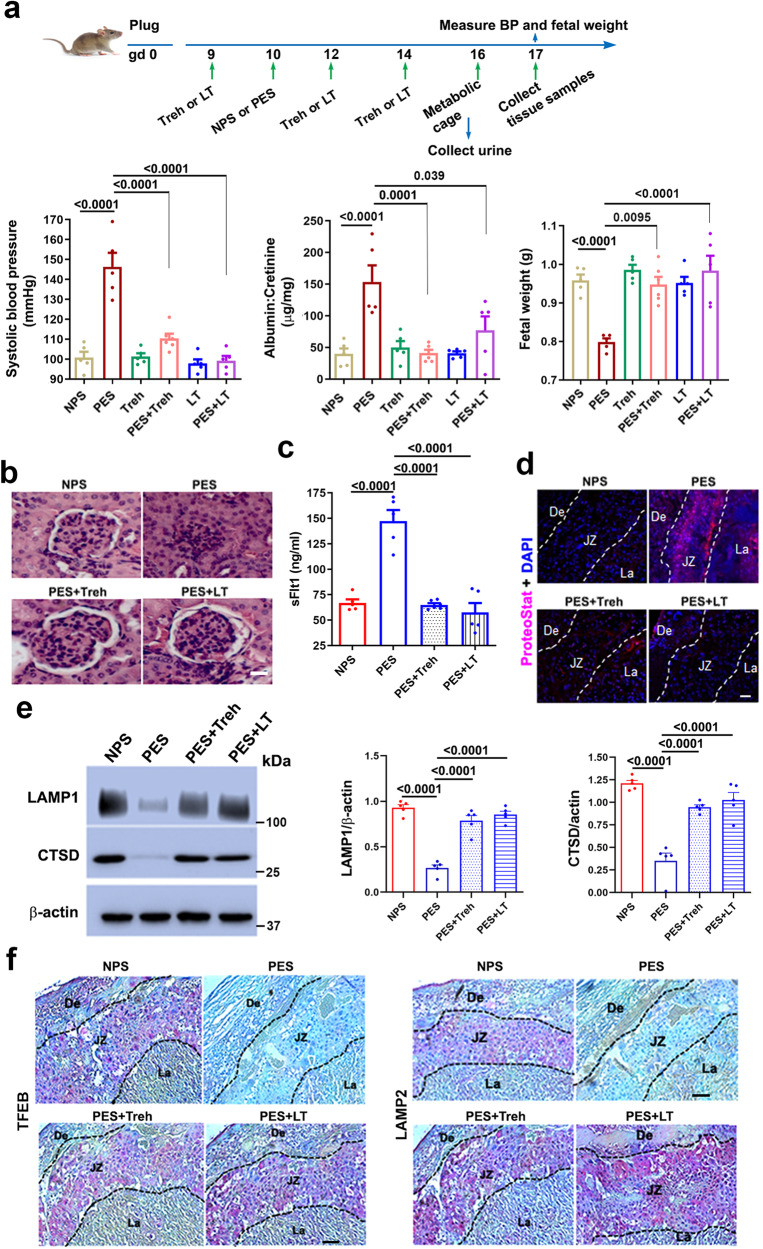
Administration of trehalose (Treh) or lactotrehalose (LT) alleviated PE-like features in a humanized mouse model of PE. **a** The top panel is a schematic diagram depicting the strategy used for the mouse model of PE and Treh/LT treatment. On gd 10, IL-10^−/−^ mice were injected with 100 µl of normal pregnancy serum (NPS) or severe PE serum (PES). Treh or LT (2 g/kg) was i.p. injected at different gds as indicated. Mice were kept in metabolic cages on gd 16 for 24 h to collect urine. Blood pressure was measured before collection of serum and tissue samples on gd 17. The lower panel shows the animal study results. Treh or LT normalizes PES-induced high systolic blood pressure (left), increases proteinuria (middle), and decreases fetal weight (right) (*n* = 5 or 6). **b** Representative H&E staining images of kidney tissue showing that Treh or LT rescued glomerular injury. **c** ELISA analysis demonstrated that Treh or LT decreased the serum sFlt-1 concentration in mice with PE (*n* = 5). **d** Treh or LT reduced protein aggregation (as detected by ProteoStat staining) in the junctional zone of the placenta in mice with PE (*n* = 5). **e** Treh or LT restored the expression of LAMP1 and cathepsin D (CTSD) in the placentas of mice with PE (*n* = 4). **f** Representative immunohistochemical images showing that Treh or LT rescued the expression of TFEB or LAMP2 in the placentas of mice with PE. De decidua, JZ junctional zone, La labyrinth. The data are presented as the means ± SEMs and were analyzed using one-way ANOVA with multiple comparisons. Bars: 40 µm.

To evaluate whether trehalose-like disaccharides target impaired autophagy in vivo and protect against the onset of PE, we injected pregnant mice i.p. with serum samples from individuals with either normal pregnancy (NPS) or severe PE (PES) on gestational day (gd) 10 combined with the administration of trehalose or lactotrehalose (2 mg/kg) on gd 9, gd 12 and gd 14. All mice were processed individually for urine collection in metabolic cages on gd 16 and blood pressure readings and tissue collection on gd 17 (see details in Fig. 5a). Our data, using a sizeable cohort of pregnant mice (*n* = 5 or more), demonstrated the ability of trehalose and lactotrehalose to normalize elevated blood pressure, proteinuria, and fetal growth restriction in mice with PE-like features (Fig. 5a).

In these experiments, both trehalose and lactotrehalose had no detrimental effects when they were administered with normal pregnancy serum (NPS) (Fig. 5a, b). Moreover, as shown in Fig. 5b, PES also caused the narrowing or disappearance of Baumann’s capsule and capillary occlusion, features of glomerular endotheliosis. These pathological changes were reversed by trehalose or lactotrehalose treatment (Fig. 5b). Trehalose and lactotrehalose also suppressed the induction of serum sFlt1 in mice with PE (Fig. 5c) and inhibited the accumulation of ProteoStat^+^ protein aggregates, particularly in the JZ of the mouse placenta (Fig. 5d). Mechanistically, both trehalose and its lacto analog increased the expression of LAMP1 and CTSD, which were significantly lower in the placentas of PES-induced mice with PE than in those of NPS-treated (non-PE) mice (Fig. 5e). Similarly, immunohistochemical analyses suggested that TFEB and LAMP2 were reduced in mice with PE but not in NPS-treated mice, and the levels of these proteins were restored by both trehalose and lactotrehalose (Fig. 5f).

To compare treatment effects based on our initial data, we used trehalose as a representative disaccharide in different experimental settings (see Fig. 5a). We established seven different treatment groups for trehalose injection: NPS + vehicle (on gd 10), PES + vehicle (on gd 10), NPS or PES (on gd 10) + trehalose (on gd 9, gd 12 and gd 14, or all 3 days), or PES (on gd 10) + maltose (on all 3 days). Trehalose given on gd 9 as a drug for prevention or gd 12 and gd 14 as a treatment modality inhibited the onset of PE-like features, including elevated blood pressure, proteinuria, fetal growth restriction, glomerular endotheliosis, and elevated sFlt1 (Supplementary Fig. 6a–d). Maltose administered at the same dose had no preventive effects on PE-like features. To determine whether renal injury induced by PES is associated with the deposition of protein aggregates, as in the placenta in pregnant mice, we assessed protein aggregates in the kidney tissue and detected substantial aggregation (Supplementary Fig. 6e). In addition, trehalose given on gd 9 or gd 12 and gd 14 rescued the expression of LAMP1 and CTSD in mice with PE (Supplementary Fig. 6f). Moreover, injection of PES in pregnant mice significantly decreased the area of the JZ of the placenta. Treatment with trehalose significantly improved the morphological appearance and size of the JZ region but had no significant effect on the area of the La (Supplementary Fig. 7a–c). In addition, the litter size was recorded, and there were no significant differences between the untreated and trehalose/lactotrehalose-treated groups of mice with PE (Supplementary Fig. 8). No fetal absorption was observed in *the* trehalose- or lactotrehalose-treated groups.

### Trehalose and lactotrehalose drive the protective effects of PE by normalizing the PE-associated transcriptome in the mouse placenta

**Fig. 6:**
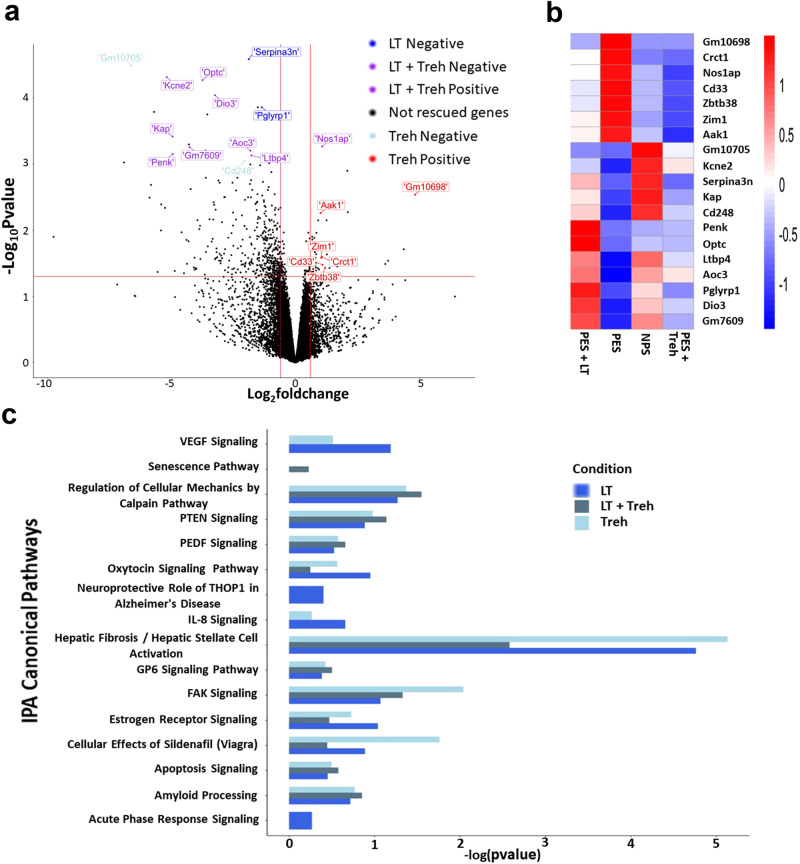
Alteration of the transcriptome in the placentas of NPS-treated (NP mice) or PES-treated mice (PE mice) subjected to various treatments. **a** Volcano plot of genes differentially expressed in the placenta from the indicated groups. The log fold change of PES versus NPS is represented on the x-axis. The y-axis shows the log10 of the *p* value. A *p* value of < 0.05 and a fold change of 2 are indicated by the red lines. The colored dots represent the top “rescued” genes that were rescued by only lactotrehalose (LT) or trehalose (Treh) or by both LT and Treh. Treh Positive: PES significantly increased expression, and Treh normalized the expression levels. Treh Negative: PES decreased expression, and Treh reversed the expression to normal levels. LT Positive and Negative or Treh + LT-positive and LT-negative were defined similarly to Treh. **b** Heatmap of the top “rescued” genes with the expression shown for the different treatment comparisons (Nas1ap, also called CAPON). **c** Ingenuity pathway analysis (IPA) showing various signaling pathways enriched in “rescued” genes. The x-axis shows negative logarithmic enrichment *p* values for all associated pathways containing the genes rescued by only LT (blue) or Treh (light blue) and both LT and Treh (gray).

To further elucidate the mechanism of the protective effects of trehalose and lactotrehalose, we performed RNA-seq analysis using placental tissue from pregnant mice exposed to the following treatments: (1) normal pregnancy serum (NPS) + vehicle, (2) preeclampsia pregnancy serum (PES) + vehicle, (3) PES + trehalose, and (4) PES + lactotrehalose. NPS or PES was injected on gd 10; vehicle or trehalose (Treh)/lactotrehalose (LT) was injected on gd 9, gd 12, and gd 14. Total RNA was extracted from the whole placenta and subjected to RNA-seq as described in the Methods section. We identified genes that were differentially expressed (*p* < 0.05) when comparing PES vs. NPS (*n* = 556), PES vs. PES + Treh (*n* = 2708), and PES vs. PES + LT (*n* = 967). Our goal was to identify “rescued” genes, i.e., genes for which PES altered expression and treatment with Treh and/or LT returned expression to almost normal levels. We identified 92 genes for which PES significantly decreased expression and Treh rescued the expression levels (PES vs. NPS, *p* < 0.05; PES vs. PES + Treh, *p* < 0.05). Six genes were identified for which PES increased expression and Treh reversed the level of expression back to almost normal levels (Supplementary Table 3). Similarly, PES decreased the expression of 106 genes, which was reversed by LT. LT also reversed the increase in expression of 1 gene caused by PES (Supplementary Table 3). Seventy-eight genes were “rescued” by both Treh and LT (Supplementary Table 3). Figure 6a shows the DEGs between the PES and NPS groups. The “rescued” genes are identified in a color-coded pattern. Figure 6b is a heatmap of the expression of the top “rescued” genes, with the expression shown for the different treatment comparisons (Fig. 6b).

To gain mechanistic insights, we performed pathway and ontology analyses of the identified “rescued” genes. Ingenuity Pathway Analysis (IPA) (Qiagen, Inc.)^43^ identified several significant pathways, including hepatic fibrosis signaling, FAK signaling, RhoA signaling, ILK signaling and integrin signaling pathways, which were enriched for genes “rescued” by Treh (*p* ≤ 10^−5^). Many of these pathways were also enriched for genes rescued by lactotrehalose (Supplementary Table 4). Gene Ontology analysis revealed enrichment in biological processes, including extracellular matrix organization, protein kinase synthesis pathways, and regulation of cell motility and migration (Supplementary Table 4). Several significant and interesting pathways identified by pathway analysis of the DEGs in the PES vs NPS comparison are shown in Fig. 6c. The *p* values for the enrichment of these pathways for the identified “rescued” genes are also plotted.

**Fig. 7:**
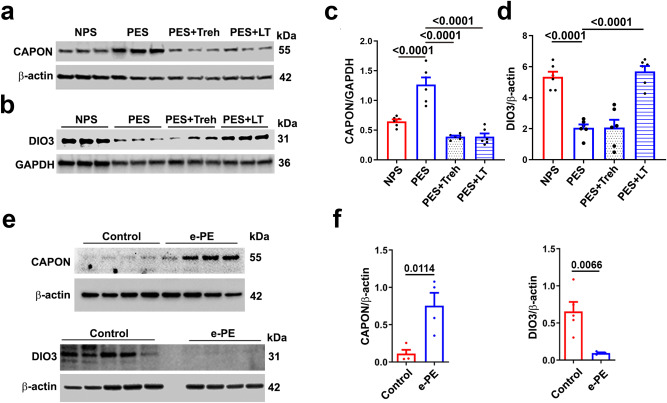
Validation of the genes identified by RNA-seq. CAPON and DIO3 from the heatmap were selected for validation experiments using NP and PE mouse placentas or human PE placentas and controls. **a**–**d** and (**e**) Western blotting analysis of CAPON and DIO3 in different groups of mouse placenta (**a**, **b**) (*n* = 6) or human e-PE placenta and control placenta (**e**) (*n* = 4–5) and quantified by ImageJ (**c**, **d**, **f**). The data are presented as the means ± SEMs and were analyzed using one-way ANOVA coupled with multiple comparisons (**c**, **d**) or Student’s *t* test (**f**). e-PE early-onset PE.

We validated several of the genes shown in Fig. 6b by evaluating their protein products by Western blotting. For comparative Western blotting analysis, we also used placental tissue from patients with early-onset severe human PE (e-PE) (demographic information is provided in Supplementary Table 2). We analyzed the levels of nitric oxide synthase 1 adapter protein (Nos1ap, also called carboxy-terminal PDZ ligand of neuronal nitric oxide synthase protein (CAPON)) and the enzyme iodothyronine deiodinase type 3 (DIO3) (Fig. 7). For CAPON, its expression was induced in the placenta of mice with PE (PES), and Treh and LT normalized the levels observed in the normal pregnant mouse placenta (NPS) group (Fig. 7a, c). In the case of DIO3, its expression was significantly reduced in the PE mouse placenta. Treh and LT reversed this trend (Fig. 7b, d). A similar expression pattern of these two proteins was observed in placental tissue from e-PE pregnancies and control pregnancies in humans (Fig. 7e, f), suggesting similar regulation of these genes in human and mouse preeclamptic placentas.

**Fig. 8:**
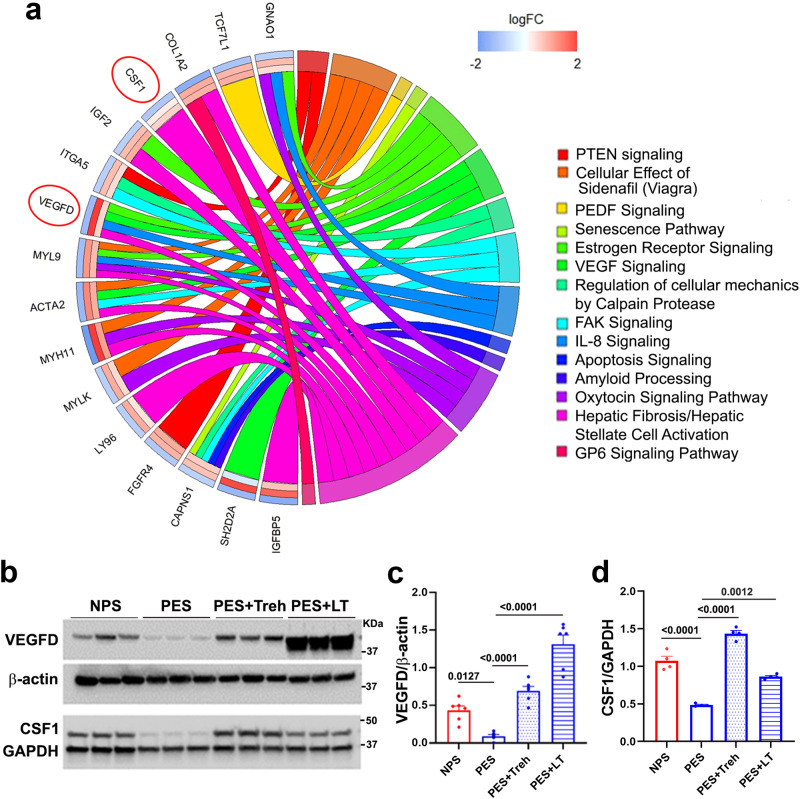
Pathway analysis of RNA-seq-identified rescue genes and gene validation for VEGF-D and CSF-1. **a** Chord plot representing the enriched pathways associated with the rescue genes. The left side of the chord plot contains the names of the genes rescued by either 50 mM LT or Treh in the enriched IPA pathways. The log FC values of each gene for three comparisons are shown as bands: PES vs. NPS (outermost), LT vs. PES (middle), and Treh vs. PES (innermost). Genes are connected with colored edges corresponding to the pathways for which they exist. **b** Validation of gene expression levels in mouse placenta using Western blotting (*n* = 6 for VEGFD, *n* = 4 for CSF1). **c**, **d** Ratios of VEGF D/actin and CSF-1/actin expression. The data are presented as the mean ± SEM and were analyzed with one-way ANOVA.

Figure 8a is an additional representation of these pathway data, displaying these enriched pathways with edges connecting the pathways and the “rescued” genes and the associated fold changes for each treatment comparison. We selected the angiogenic factor VEGF-D and colony-stimulating factor 1 (CSF-1) for validation by Western blotting (Fig. 8b–d). These results suggested that VEGFD and CSF-1 were inhibited in the preeclamptic mouse placenta and confirmed that Treh and LT independently restored the protein levels of VEGF-D and CSF-1 (Fig. 8b–d).

## Discussion

We and others have shown that PE is associated with proteinopathy^9–16,44–46^. We suggest that proteinopathy is associated with impaired autophagy in PE^9–16^. Here, we show that the administration of trehalose and its trehalase-resistant lacto analog, lactotrehalose, provides therapeutic benefits in a humanized mouse model of PE. The mechanistic basis for the effects of these two disaccharides is the normalization of impaired autophagy and inhibition of proteinopathy. These effects were demonstrated in a cellular model of PE using primary human trophoblasts as well as the placenta of mice with PE. Furthermore, trehalose and lactotrehalose reversed many of the PE-associated transcriptomic changes in placental tissue from mice with PE.

The search for therapeutic approaches for PE is ongoing. Although a reduction in circulating sFlt1 has been attempted and clinical trials are underway^47^, no definite treatment is yet available. Recently, next-generation therapeutic options have been reported. RNAi-based therapy has been proposed to target sFlt1 in a baboon model of PE, which effectively alleviated some symptoms of this severe pregnancy complication with no side effects on the fetus^48^. However, no therapy has yet targeted upstream regulatory events that lead to placental insufficiency and elevated production of sFlt1-like molecules. By targeting impaired autophagy and proteinopathy in PE-associated cellular and in vivo models, we provide evidence that these nonmammalian disaccharides provide a significant therapeutic benefit in these experimental models of PE.

The development of predictive assays and effective therapeutic options for PE has been hampered by our poor understanding of its etiology. Our recent observations that PE is associated with impaired autophagy, the accumulation of protein aggregates in the placenta and circulation^9–16^, and sterile inflammation^25^ suggest a novel therapeutic approach for PE. We have also reported that H/R induces the formation of protein aggregates in primary human trophoblasts isolated from placenta samples from normal pregnancy^13^. The etiological role of impaired autophagy and proteinopathy in PE has been validated by using conditional autophagy-deficient trophoblast cell lines or pregnant mouse models overexpressing human proteins or depleted of autophagy-related proteins. In the setting of impaired autophagy in the preeclamptic placenta, we demonstrated impairment of the lysosomal biogenesis machinery, including significantly reduced expression of LAMP1, LAMP2, and CTSD and inhibited nuclear translocation of TFEB, the master transcriptional regulator of lysosomal biogenesis-related proteins^2,11,26^. Similar results were obtained in a study of the placenta from PE model mice induced by the administration of serum from pregnant women with severe PE. Under conditions of proteinopathy in the serum and human placenta in PE, we have demonstrated the presence of aggregated TTR, amyloid β, and *cis* P-tau proteins in the serum of patients with early- or late-onset severe PE^16^. These observations suggest that these new mechanistic insights can lead to novel therapeutic options for PE.

Although trehalose is considered safe, we included the trehalase-resistant analog lactotrehalose in our analysis to assess its ability to restore autophagy and inhibit proteinopathy. Lactotrehalose (α-D-galactopyranosyl-(1,1)-α-D-glucopyranoside) differs from trehalose only in the orientation of a hydroxyl group at carbon 4 (Supplementary Fig. 4a)^33^. To date, lactotrehalose has been characterized only for its resistance to trehalase and for its ability to increase energy metabolism without promoting any gut microbial infection in rodents^33^. Trehalose has been found to be clinically favorable even in phase 2 trials as a potential drug for treating human diseases associated with neurotoxic misfolded and aggregated proteins, including AD^49^, Parkinson’s disease^29^, spinocerebellar ataxia^50^, ALS^31,32^ and oculopharyngeal muscular dystrophy^51^. A key feature of trehalose is its ability to function as a chemical chaperone that assists in refolding and stabilizing denatured proteins^52^. Since PE is a disorder of impaired autophagy and proteinopathy, our focus on trehalose-like disaccharides is well justified for the treatment of a pregnancy disorder such as PE, which has not been fully elucidated. Trehalose and lactotrehalose, but not maltose, sucrose or D-glucose, were found to inhibit protein aggregation or restore the TFEB-based autophagy machinery. TFEB regulation is restored by trehalose and lactotrehalose via the dephosphorylation of the calcium-dependent phosphatase calcineurin and the subsequent nuclear translocation of TFEB. This finding agrees with our previous observations on calcineurin-mediated nuclear translocation of the transcription factor NFAT^38,39^.

Using TEM, we demonstrated that trehalose mediates the restoration of ultrastructural features, including autophagosomes, autolysosomes, and mitochondria disrupted and damaged by H/R, in primary human trophoblasts. These observations are important because trehalose-like saccharides not only restore the lysosomal machinery but also repair ultrastructural features damaged by triggers such as hypoxia.

RNA-seq analysis of placental tissue from treated and untreated mice demonstrated that compared with normal pregnant mice, mice with PE had an altered transcriptomic profile. Importantly, treatment of mice with PE with trehalose or lactotrehalose restored the transcriptomic profile, with the identification of many genes that showed “rescued” expression, i.e., the normalization of expression levels to that of the mice with a normal pregnancy. Additionally, pathways that were enriched in genes that were rescued by trehalose and/or lactotrehalose were identified. These pathways all have established connections and play a role in multiple biological processes that regulate autophagy, metabolism, apoptosis, embryonic development, signal transduction, and transcriptional activity. For example, the “rescued” genes were found to be significantly enriched in the RhoA signaling pathway. Aberrant RhoA signaling has been identified in neurodegenerative diseases such as AD, which shares its etiology with PE^53,54^. Pathways associated with fibrosis were also identified. A previous study showed that autophagy enhancers mitigate several fibrosis-related diseases^55^. Fibrosis is also a prominent pathological feature of preeclamptic placentas, where evidence suggests that it is controlled by TGF-β signaling^56^. Focal adhesion kinase (FAK) signaling is another identified target. FAK is a known key mediator of human trophoblast and early placental development, and there is evidence of links between FAK complexes and autophagy regulation^57^. Identification of these pathways provides insight into the mechanism by which trehalose and its lacto analog, by targeting the autophagy pathway and protein aggregation, can effectively ameliorate PE.

We validated several key genes and pathways that were differentially expressed in mice with PE, such as the CAPON and DIO3 genes and the angiogenic and immune regulation pathways represented by VEGF-D and CSF-1. CAPON is a *tau*-binding protein and has previously been shown to induce protein aggregation and neurodegeneration^58^. DIO3 catalyzes the release of iodine directly from thyroxine hormones, which convert the thyroid hormones thyroxine (T4) and 3,3′,5-triiodothyronine (T3) to their inactive metabolites 3,3′,5′triiodothyronine and 3,3′-diiodothyronine, respectively, and has been shown to be highly expressed in human trophoblasts and plays a critical role in maintaining normal pregnancy^59^. Our results suggest that increased CAPON and decreased DIO3 may be associated with the pathogenesis of PE. Additionally, while the immunomodulatory cytokine CSF-1 was enriched in the fibrosis pathway, this molecule is also involved in the PI3K-AKT and RAP1 signaling pathways, which are known mediators of PE^60^. Our validation data confirmed that trehalose and lactotrehalose normalize the gene and protein expression of these factors to the levels found in normal pregnant mice.

Our present study demonstrated that impaired autophagy and proteinopathy play important etiological roles in PE and can be targeted by trehalose-like disaccharides for potential therapeutic intervention. However, the potential reproductive toxicity of any drug during pregnancy is extremely important. Thus, the clinical acceptance of trehalose-like disaccharides for the treatment of PE will depend on further evaluation of overall toxicity, teratogenicity, and safe routes of administration. Notably, our data in pregnant mice did not show any adverse effects of trehalose or lactotrehalose on normal pregnancy outcomes, including litter size, placental morphology, or fetal weight. Thus, we believe that drugs with demonstrated efficacy, such as trehalose, hold great promise and deserve further evaluation in patients with PE.

### Supplementary information

Supplementary Information

Supplementary Table 3

Supplementary Table 4

## Data Availability

The RNA sequencing data discussed in this publication have been deposited in the NCBI Gene Expression Omnibus and are accessible through the GEO Series accession number GSE202129.

## Supplementary information

The online version contains supplementary material available at 10.1038/s12276-024-01234-x. Supplementary information accompanies the manuscript on the Experimental & Molecular Medicine website (http://www.nature.com/emm/).
