# Supplementary material for: Exploiting sweet relief for preeclampsia by targeting autophagy-lysosomal machinery and proteinopathy
Source: Exp Mol Med. 2024 May 17;56(5):1206–20. doi: 10.1038/s12276-024-01234-x (PMC11148015; doi:10.1038/s12276-024-01234-x)
Supplement: Supplementary file 1 — Supplementary Information [file 12276_2024_1234_MOESM1_ESM.pdf]

Supplementary Information:

Huang et al, Supplementary Fig. 1

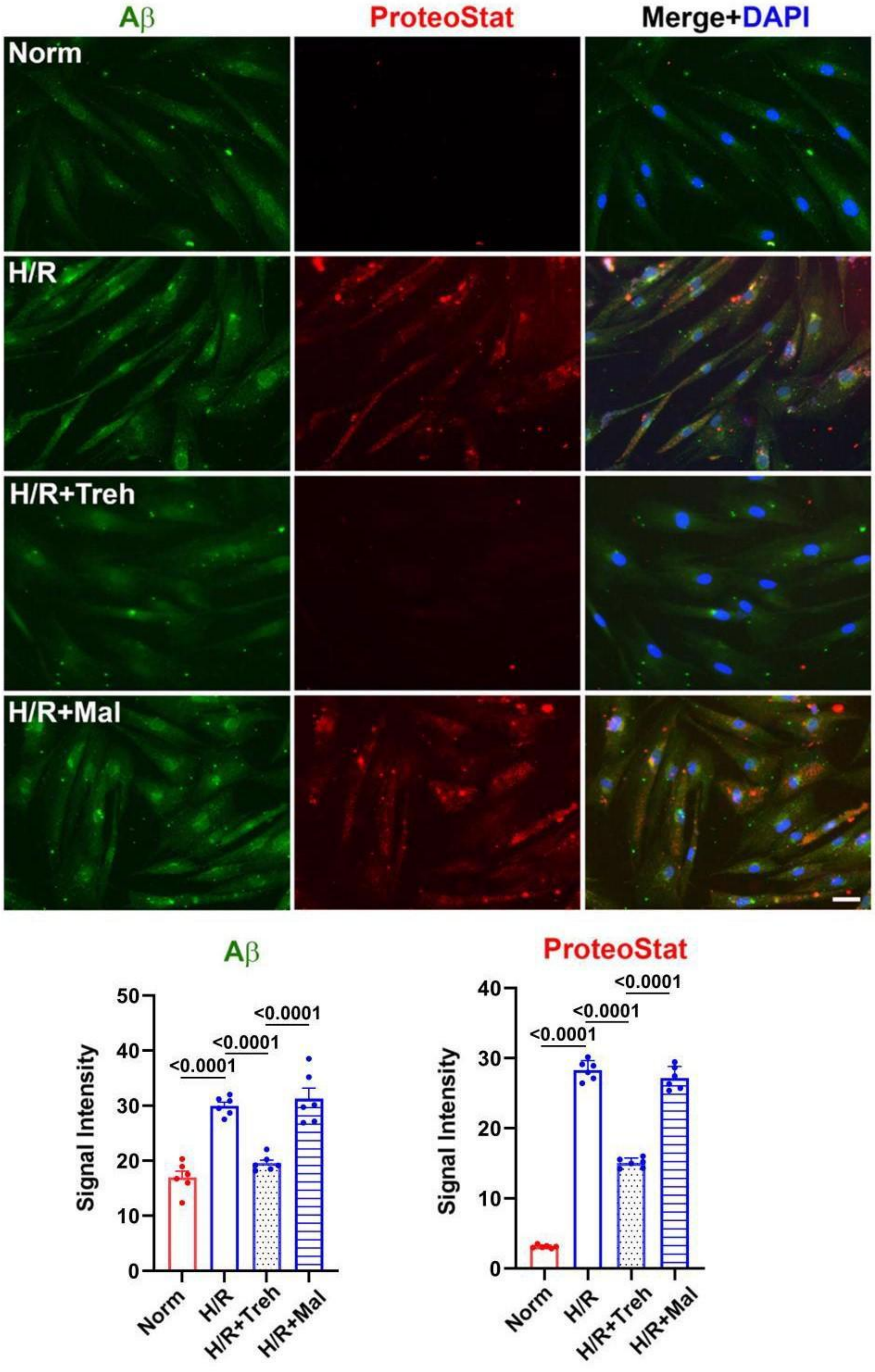

Supplementary Fig. 1. Trehalose, not maltose, reduces H/R-induced accumulation of beta-amyloid in primary human trophoblasts. Cells were treated with normoxia or H/R for 3 days with or without the presence of 50 mM of maltose (Mal) or trehalose (Treh), immunostained for beta-amyloid (A , green), and counterstained with ProteoStat dye (red). The nuclei were stained with DAPI. The intensity of A and ProteoStat signals was quantified using ImageJ. Data were presented as mean ± SEM, and statistical analysis was performed by two-way ANOVA corrected for multiple comparisons. Bar: 20mm.

Huang et al. Supplementary Fig. 2

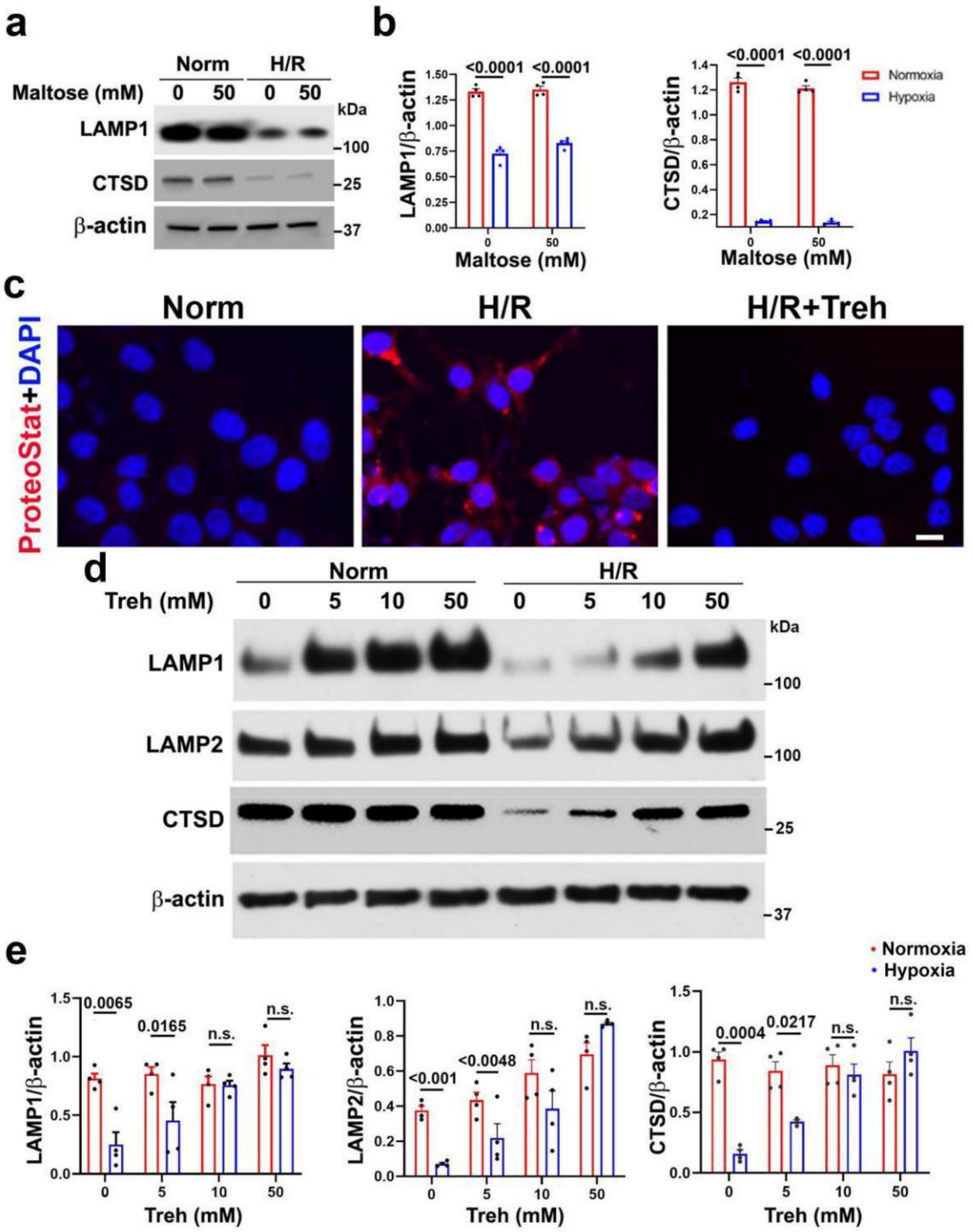

Supplementary Fig 2. Maltose fails to alter lysosomal biogenesis in primary human trophoblasts, and Trehalose rescues H/R-induced impairment of autophagy in the human TCL1 trophoblast cell line. Cells were exposed to normoxia or H/R for 3 days with or without the presence of 50 mM of maltose (Mal) or trehalose (Treh). Then, cells were lyzed and subjected to Western blot analysis or fixed for immunofluorescence staining. a, b Maltose did not significantly change the expression levels of LAMP1 and cathepsin D (CTSD) in primary human trophoblasts exposed to normoxia (Norm) or H/R (n=4). c, Representative ProteoStat staining images show that trehalose (50 mM) inhibited protein aggregation in TCL1 cells exposed to H/R (n = 4). d-e Western blot (d) and statistical analysis (e) show that trehalose reversed H/R-induced downregulation of LAMP1, LAMP2, and CTSD abundance in TCL1 cells in a dose-dependent manner (n = 4). Data in b, e were presented as mean ± SEM, and statistical analysis was performed by two-way ANOVA corrected for multiple comparisons. n.s.: not significant.

Huang et al. Supplementary Fig. 3

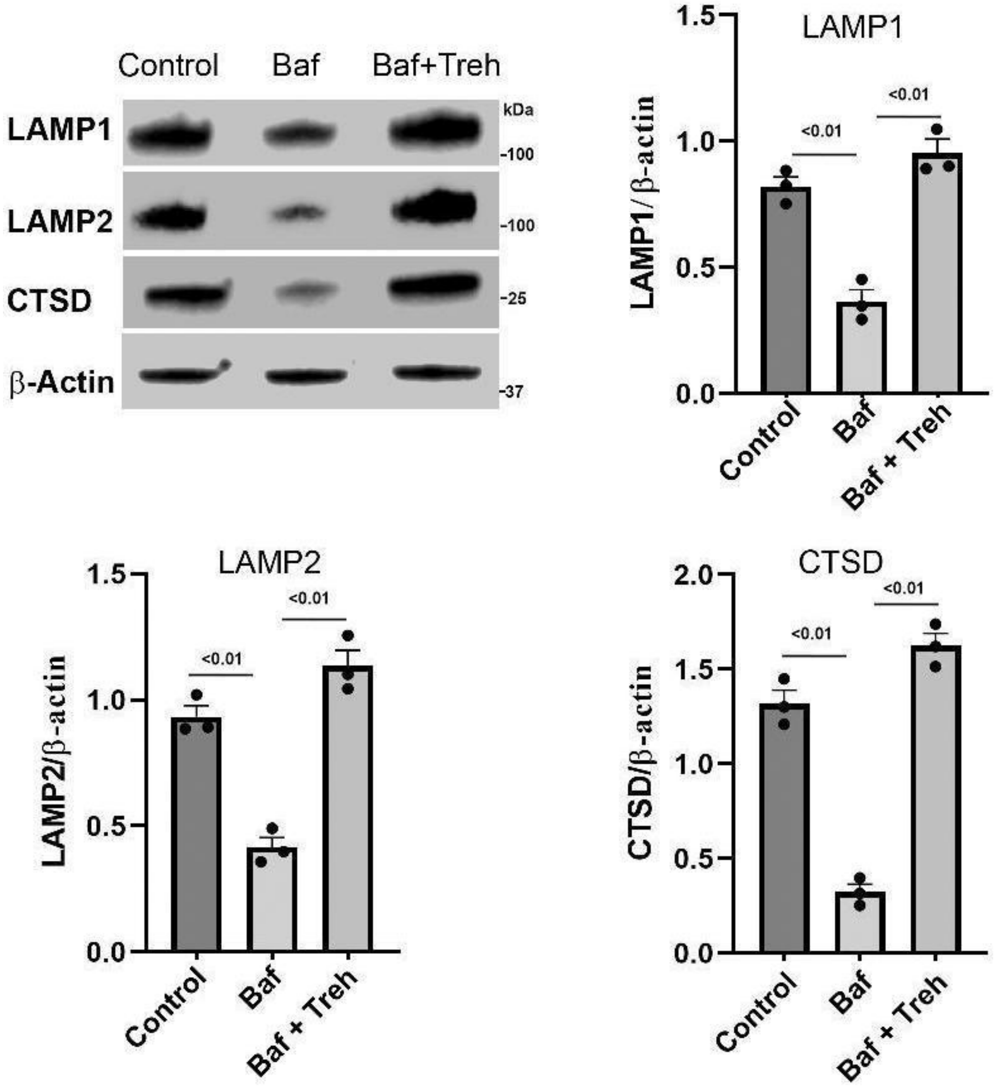

Supplementary Fig. 3 Bafilomycin inhibits the lysosomal biogenesis protein, which can be rescued by trehalose. Primary human trophoblasts were treated with 1 of bafilomycin A or vehicle overnight, then washed with PBS three times. Fresh media was added with or without 50mM of Treh. Cells were cultured for another 24hr before cell collection for Western blot analysis. Representative images showed that Bafilomycin treatment significantly inhibited LAMP1, LAMP2 and Casthepsin D (CTSD), and trehalose rescued their expression . Data are presented as mean ±SEM, and statistical analysis was performed by One-way ANOVA analysis corrected for multiple comparisons ($n= 3$, $p < 0.01$).

Huang et al, Supplementary Fig. 4

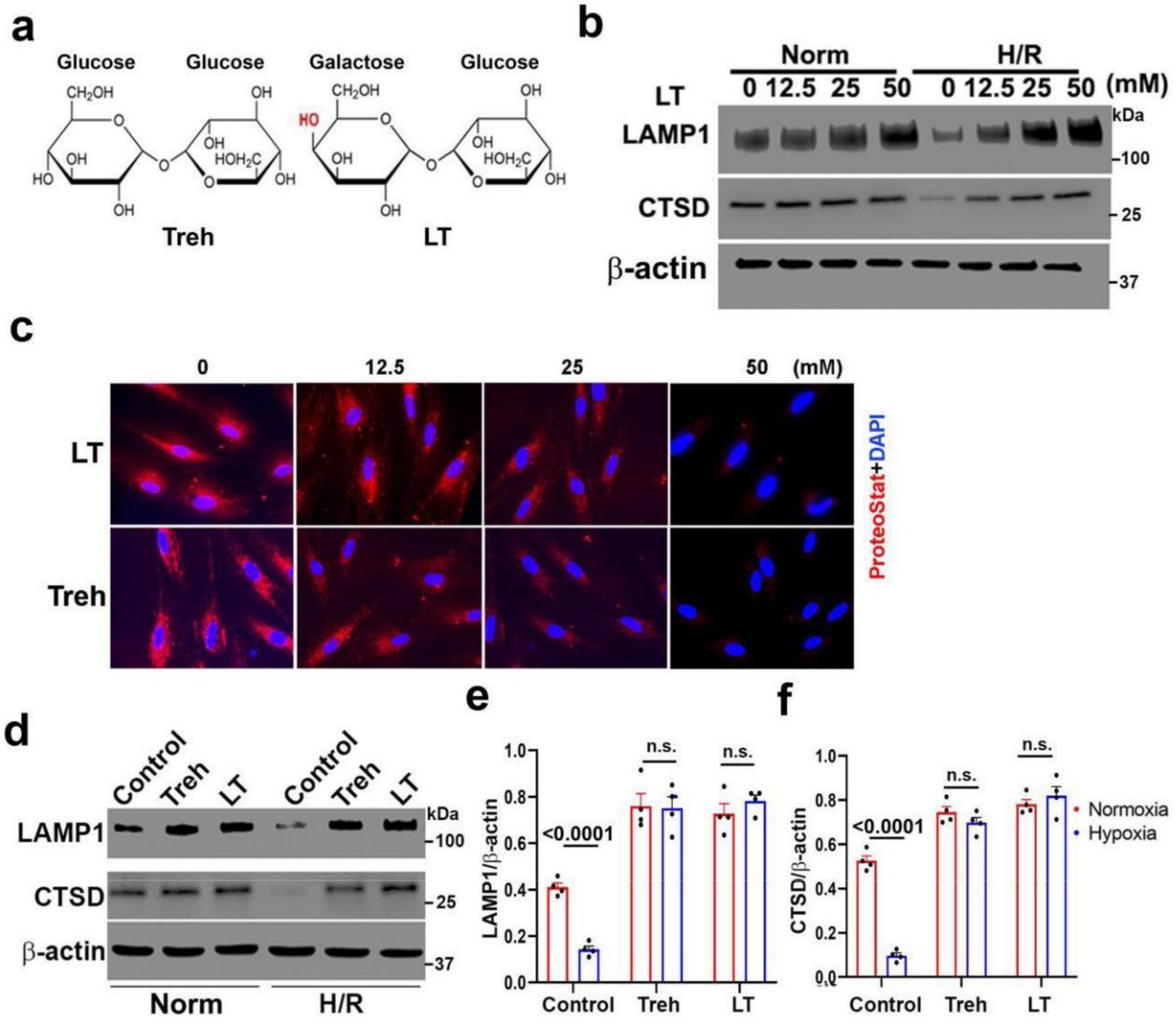

Supplementary Fig. 4 Lactotrehalose and trehalose are equally potent in restoring H/R-induced inhibition of lysosomal biogenesis proteins and inhibiting the accumulation of protein aggregates in primary human trophoblasts (PHTs). PTHs were exposed to normoxia or H/R for 3 days with or without treatment of 50 mM of lactotrehalose (LT) or trehalose (Treh). Then cells were lyzed for Western blotting; or fixed for ProteoStat staining. a Molecular structure of Treh (two glucoses) and LT (one glucose and one galactose). b LT rescued the levels of LAMP1 and cathepsin D (CTSD) in H/R-treated primary human trophoblasts in a dose-dependent manner (n = 4). c Representative ProteoStat staining images demonstrate that LT, like Treh, dose-dependently inhibited protein aggregation in H/R-exposed trophoblasts. d-f Western blot (d) and statistical analysis show that LT, like Treh (at 50mM), restored the expression levels of LAMP1 and CTSD in TCL1 cells exposed to H/R. Data are presented as mean ± SEM in e and f, and statistical analysis was performed by two-way ANOVA corrected for multiple comparisons. n.s.: not significant, (n = 4).

Huang et al. Supplementary Fig. 5

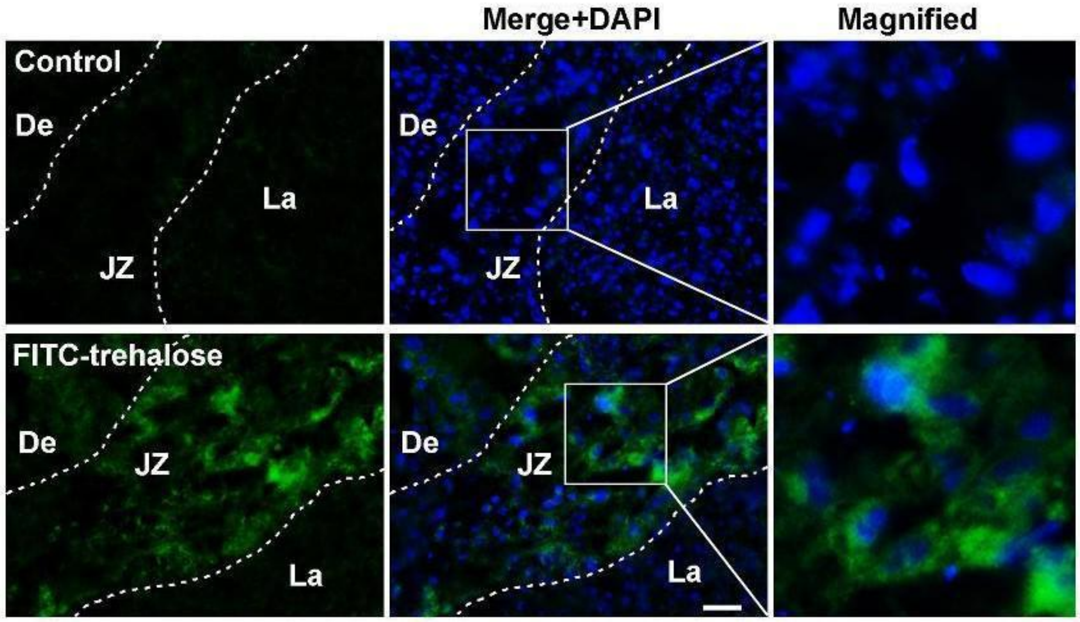

Supplementary Fig. 5. Evidence for trehalose transport into the mouse placenta post 6 hrs i.p. injection. Mice at gd 14 were i.p. injected with 0.1 mg of FITC-trehalose or vehicle and sacrificed after 6 hrs of administration. Frozen sections of the placental tissue were observed under a fluorescent microscope. Representative images showed a strong fluorescent green signal for trehalose in the placenta in the areas of De and JZ from mice injected with FITC-trehalose, no green signal from control (n = 4). Boxed areas were magnified as shown in the right column. De: decidual zone, JZ: junctional zone, La: Labyrinth zone. Bar: 20 m.

Huang et al, Supplementary Fig. 6

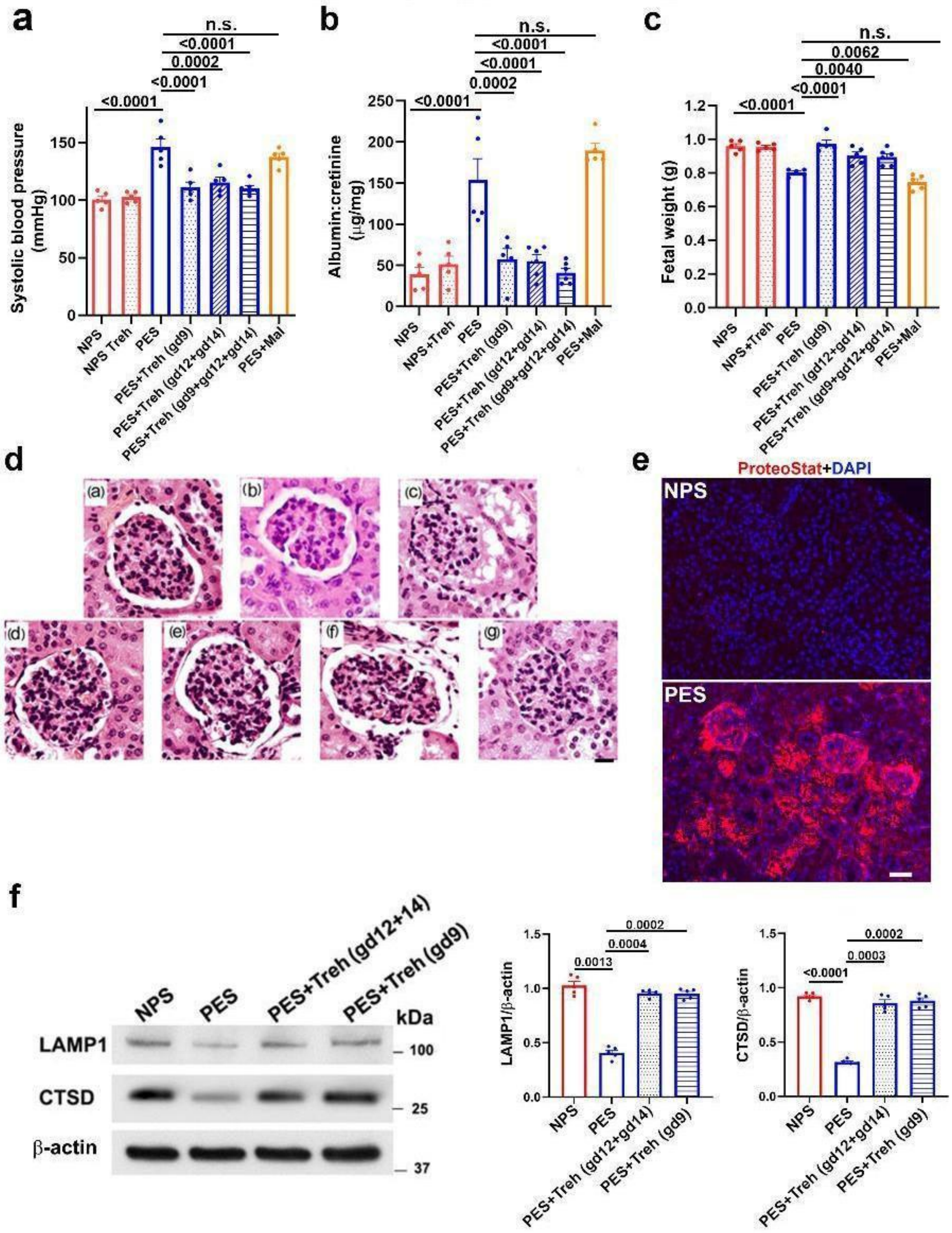

Supplementary Fig. 6. Trehalose, not maltose, rescues PE-like features in a humanized mouse model. a-c Effects of trehalose (Treh, 50mM) or maltose (Mal, 50mM) administration on the systolic blood pressure (a), proteins in urine (albumin:cretinine) (b), and fetal weight (c) in PE mice vs. control mice (n=5-6). d Representative HE staining images show the morphological structure of glomeruli in the kidney from mice injected with (a) NPS, (b) NPS + Treh, (c) PES, (d) PES +Treh (gd 9), (e) PES +Treh (gd 12, gd 14), (f) PES + Treh (gd 9 + gd 12 + gd 14), or (g) Mal (gd 9 +gd 12 + gd 14). e Proteostat staining of the kidneys from mice injected with NPS and PES. n=4. Bar=45 m. f Western blotting and statistical analysis demonstrate that Treh administrated at gd 9 alone, or at gd 12 and gd 14 normalized the expression levels of LAMP1 and cathepsin D (CTSD) in PE mice. Data are presented as mean ± SEM, and statistical analysis was performed by One-way ANOVA corrected for multiple comparison (n = 5). n.s.: not significant.

Huang et al, Supplementary Fig. 7

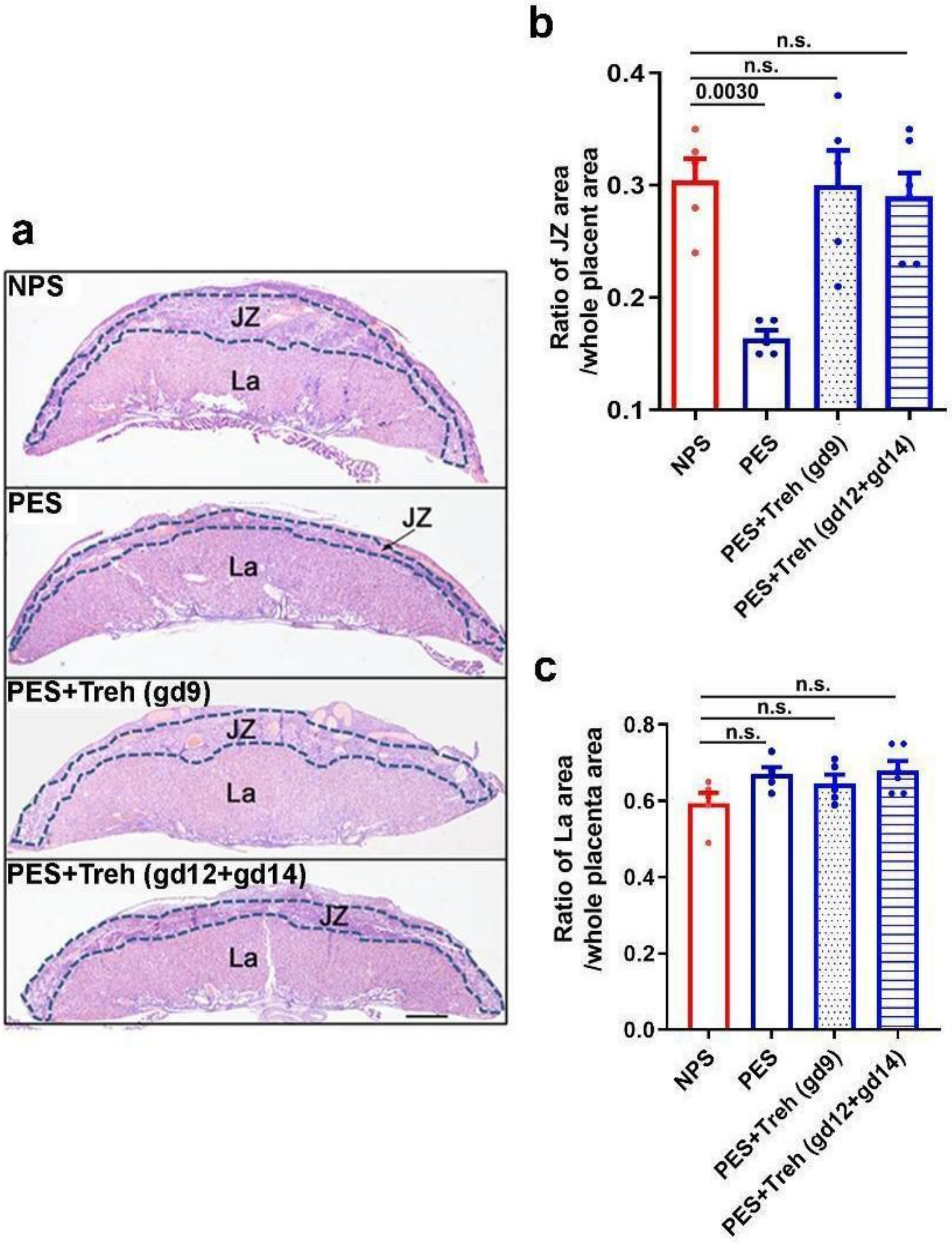

Supplementary Fig. 7. Trehalose reverses the decrease in the junctional zone (JZ) area of the placenta from PE mice. a Representative H&E staining images show the effect of trehalose (Treh) administration at gd 9 or gd 12+gd 14 on the JZ and labyrinths (La) morphological areas in the placenta from PE mice and control mice. b, c Quantitative analysis shows that Treh injection in PE mice significantly normalized the JZ area to total placental area (b), but did not show any major effect on the ratio of the La area to total placental area (c) in PE mice, suggesting that the La area was not significantly affected in PE mice. Data are presented as mean ± SEM, and statistical analysis was performed by One-way ANOVA analysis ($n = 5\text{-}6$, $p < 0.01$). n.s.: not significant. Bar: 600 mm.

Huang et al, Supplementary Fig. 8

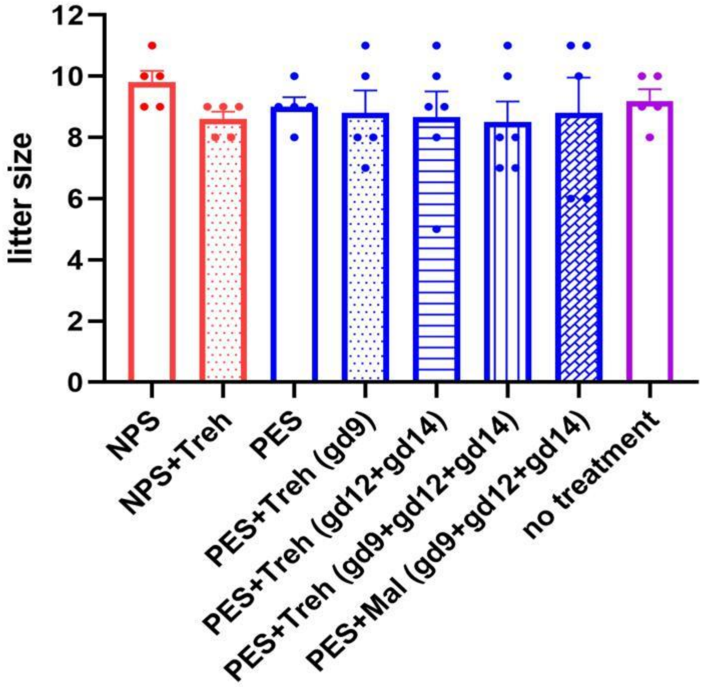

Supplementary Fig. 8 Trehalose shows no effect on litter size in normal pregnancy or PE mice. Pregnant mice were treated as indicated and did not show a significant difference in litter size. Data are presented as mean ± SEM, and statistical analysis was performed by One-way ANOVA analysis ($n = 5$-6, $p > 0.05$).

Supplemental Tables

Supplementary Table 1: Demographic and clinical characteristics of patients

Variable	Early onset preeclampsia (n=7)	Gestational age-matched control (n=7)	*p*-value
Age (years)	29.8(7.1)	23.7(5.9)	†† 0.14[1]
Gestational age at delivery (weeks)	30.8(1.7)	31.5(2.5)	†† 0.06[3]
Maximum systolic blood pressure (mmHg)	180.2(13.7)	119.3(11.5)	††<0.001[3]
Maximum diastolic blood pressure (mmHg)	113.1 (6.3)	73.5(3.8)	††<0.001[3]
AST (U/L)	226.3 (304.4)	11 – 30*	N/A
Serum creatinine (mg/dl)	0.98 (0.27)	0.5 – 1.1*	N/A
Urine protein: creatinine	5.0 (6.6)	< 0.3*	N/A
Birth weight (grams)	1334(129)	2133(382)	††<0.001[1]

Data presented as Mean (standard deviation) for continuous variables Data presented as n (%) for categorical variables

*AST, serum creatinine, and urine protein:creatinine not measured in Control subjects and presented as normal ranges.

[1] t-test

[2] Fisher's exact test

[3] Wilcoxon rank-sum

†† early onset preeclampsia with severe features versus gestational age-matched controls

Supplementary Table 2：List of Antibodies

Antibodies were purchased from commercial sources. Here are the specifics of their application.

Antibody	Vendor	Catalogue#	Dilution
β actin mouse mAb	Abcam	ab6276	1:5000 (Western blotting)
LAMP2 rabbit Ab	Invitrogen	PA1-655	1:50 (Immunohistochemistry (IHC)
CAPON mouse mAb	Santa Cruz Tech	sc-374504	1:500 (Western blotting)
Amyloid mouse mAb	Abcam	Ab11132	1:200 (Immunofluorescence)
Human Transthyretin hTTR	Dako	A0002	1:50 (Immunofluorescence)<br>1:500 (Western blotting)
LAMP1 rabbit Ab	Cell signaling	9091	1:1000 (Western blotting)
LAMP2 mouse mAb	Abcam	Ab25631	1:1000 (Western blotting)
SERPINA3 rabbit Ab	Abcam	Ab205198	1:1000 (Western blotting)
TFEB rabbit Ab	Proteintech	13372-1-AP	1:200 (IHC)<br>1:1000 (Western blotting)
Phosph TFEB rabbit Ab	Millipore	ABE1971	1:500 (Western blotting)
Cathepsin D rabbit Ab	Abcam	Ab75852	1:2000 (Western blotting)
Histone H3 rabbit Ab	Upstate	06-755	1:1000 (Western blotting)
DIO3 rabbit Ab	Invitrogen	PA5-22886	1:1000 (Western blotting)
VEGFD rabbit Ab	Abcam	Ab155288	1:1000 (Western blotting)
CSF1(M-CSF) rabbit Ab	Abcam	Ab233387	1:1000 (Western blotting)
Serpin A3N goat Ab	A&D system	AF4709	1:2000 (Western blotting)

Secondary antibodies:

Goat anti-mouse IgG-HRP conjugate (Alpha Diagnostic, 40320-200, 1:1000) Western blotting

Goat anti-rabbit IgG-HRP conjugate (cell Signaling, 7074, 1:1000) Western blotting

Bovine anti-goat IgG-HRP conjugate(Santa Cruz, sc-2350, 1:5000) Western blotting

Alex Fluor 594 donkey anti-rabbit IgG (1:500), Alex Fluor 488 donkey anti-rabbit IgG (1:500),

Alex Fluor 594 donkey anti-mouse IgG (1:500) were from Invitrogen (Immunofluorescence)

Supplementary Table 3. See separate file

Supplementary Table 3.1: Genes for which expression was disrupted by exposure to preeclampsia serum (PES vs NPS p<.05, FPKMs>1, Fold change >2) and rescued by treatment with Lactotrehalose (PE vs LT p<.05). Gene IDs, symbols, log2FC between three pairwise comparisons and associated p-values are provided for each gene.

Supplementary Table 3.2: Genes for which expression was disrupted by exposure to preeclampsia serum (PES vs NPS p<.05, FPKMs>1, Fold change >2) and rescued by treatment with Trehalose (PE vs Treh p<.05). Gene IDs, symbols, log2FC between three pairwise comparisons and associated p-values are provided for each gene.

Supplementary Table 3.3: Genes for which expression was disrupted by exposure to preeclampsia serum (PES vs NPS p<.05, FPKMs>1, Fold change >2) and rescued by treatment with both Trehalose (PE vs Treh p<.05) and Lactotrehalose (PE vs LT p<.05).

Supplementary Table 4. See separate file

Supplementary Table 4.1: Gene ontology biological processes enriched for genes for which expression was disrupted by exposure to preeclampsia serum and rescued by treatment with Lactotrehalose, using Panther Over-representation analysis.

Supplementary Table 4.2: Gene ontology biological processes enriched for genes for which expression was disrupted by exposure to preeclampsia serum and rescued by treatment with Trehalose, using Panther Over-representation analysis.

Supplementary Table 4.3: Gene ontology molecular processes enriched for genes for which expression was disrupted by exposure to preeclampsia serum and rescued by treatment with both Lactotrehalose and Trehalose, using Panther Over-representation analysis.

Supplementary Table 4.4: Ingenuity Pathway Analysis of enriched for genes for which expression was disrupted by exposure to preeclampsia serum and rescued by treatment with Lactotrehalose.

Supplemeantary Table 4.5: Ingenuity Pathway Analysis of enriched for genes for which expression was disrupted by exposure to preeclampsia serum and rescued by treatment with Trehalose

Supplementary Table 4.6: Ingenuity Pathway Analysis of enriched for genes for which expression was disrupted by exposure to preeclampsia serum and rescued by treatment with Lactotrehalose and Trehalose.
